# Non-caveolar caveolin - 1 in retinal Müller glia promotes innate immune responses

**DOI:** 10.1016/j.jbc.2026.113290

**Published:** 2026-06-23

**Authors:** Eric N. Enyong, Olawale O. Bankole, Jami M. Gurley, Mark E. McClellan, Libin Liu, Shi-Ying Ding, Martin-Paul Agbaga, Ana J. Chucair-Elliott, John D. Ash, Michael H. Elliott

**Affiliations:** 1 Department of Biochemistry & Physiology, Dean A. McGee Eye Institute, University of Oklahoma Health Campus, Oklahoma City, Oklahoma, USA; 2 Department of Ophthalmology, Dean A. McGee Eye Institute, University of Oklahoma Health Campus, Oklahoma City, Oklahoma, USA; 3 Department. of Biochemistry, Boston University School of Medicine, Boston, Massachusetts, USA; 4 Department of Cell Biology, University of Oklahoma Health Campus, Oklahoma City, Oklahoma, USA; 5 Department of Ophthalmology-UPMC Vision Institute, University of Pittsburgh School of Medicine, Pittsburgh, Pennsylvania, USA

**Keywords:** caveolae, caveolin, CAVIN1/PTRF, glial cell, innate immunity, interleukin 6 (IL-6), lipid raft, Müller glia, retina, Toll-like receptor (TLR)

## Abstract

Caveolae are specialized plasma membrane invaginations implicated in ocular diseases including primary open angle glaucoma, diabetic retinopathy and age-related macular degeneration. Caveolin - 1 (CAV1) typically functions within caveolae where it associates with a co-regulatory protein, CAVIN1 (also known as polymerase I and transcript release factor; PTRF), which is necessary for caveolae formation. However, CAV1 can also reside outside of caveolae in planar “scaffolds,” though the function of this non-caveolar CAV1 remains unclear. Here we show that Müller glia, the major macroglial cells of the retina, abundantly express CAV1 with minimal CAVIN1/PTRF expression both *in situ* and in the MIO-M1 Müller glial cell line. Transmission electron microscopy confirmed that morphologically identifiable caveolae are virtually absent in Müller glia, indicating that CAV1 is predominantly non-caveolar. Transgenic CAVIN1/PTRF expression induced caveolae formation, demonstrating functional competence. Non-caveolar CAV1 in MIO-M1 Müller glia promoted Toll-like receptor - 4 (TLR4) signaling, as either CAV1 silencing or its sequestration into caveolae by CAVIN1/PTRF overexpression significantly suppressed lipopolysaccharide (LPS)-induced interleukin - 6 (IL - 6) upregulation through reduced NF-κB activation. Conversely, in human retinal endothelial cells (HRECs) where CAV1 predominantly localizes to caveolae, CAV1 silencing enhanced inflammatory responses. These results demonstrate that non-caveolar CAV1 in Müller glia promotes a pro-inflammatory phenotype that can be attenuated by CAV1 silencing or sequestration into caveolae, suggesting cell context-specific roles for CAV1 in inflammatory regulation with potential therapeutic implications for ocular inflammatory diseases.

Caveolae are specialized 50 to 100 nm flask-shaped plasma membrane invaginations that serve as critical platforms for signal transduction, endocytosis, lipid metabolism, and mechanotransduction (1, 2, 3). Caveolins, a family of 22 to 24 kDa proteins, are critical for caveolae formation and function (4, 5) with CAV1 being essential for caveolae biogenesis in most cell types (3) and CAV3 serving this role specifically in striated and cardiac muscle (6, 7). CAV2 is not required for caveolae formation and depends on CAV1 for membrane localization and function (8). Ectopic expression of CAV1 in CAV1-deficient cells results in caveolae formation, while deletion of CAV1 causes loss of caveolae (9).

While necessary for caveolae biogenesis, CAV1/3 are not sufficient and require another protein called CAVIN1 (also known as PTRF or Polymerase I and Transcript Release Factor) (10, 11). CAVIN1/PTRF-deficient mice do not form morphologically-identifiable caveolae in any tissue (12). Several studies demonstrate that CAV1 and CAVIN1/PTRF co-regulate each other, such that deletion of one significantly downregulates expression of the other, resulting in loss of caveolae (12, 13). In tissues from global CAVIN1/PTRF knockout (KO) mice, CAV1 protein levels are significantly downregulated (12, 13) while *Cav1* mRNA is upregulated, implying a compensatory response to CAV1/caveolae instability. Overexpression of CAVIN1/PTRF in deficient cells results in a concomitant increase in CAV1 expression and stabilization, and lack of CAVIN1/PTRF destabilizes membrane-bound CAV1 and promotes its lysosomal degradation (10, 14, 15, 16). These results underscore the important co-regulatory roles of CAV1 and CAVIN1/PTRF in caveolae structure and function.

Although CAV1 and caveolae have been extensively studied, their critical role in ocular disease pathogenesis has not been fully elucidated. Variants at the *CAV1/2* gene locus are associated with risk of primary open angle glaucoma (17, 18, 19). CAV1 and caveolae also play important roles in the blood–retinal barrier function (20, 21, 22) and in retinal inflammatory modulation (23, 24, 25, 26, 27). In non-ocular tissues, CAV1 is frequently associated with regulation of innate immune signaling (28, 29), but there are opposing observations in the literature about whether CAV1 promotes or suppresses inflammation (23, 30). For example, dependent on cell type-specific context, CAV1 can either promote (31, 32, 33) or suppress Toll-like receptor - 4 (TLR4) responses (24, 34, 35). Specific deletion of CAV1 from the vascular endothelium enhances lipopolysaccharide (LPS)-induced, TLR4-mediated inflammatory responses in the lung (36). In the retina, global *Cav1* gene ablation suppresses TLR4-induced inflammatory cytokine release while paradoxically enhancing immune cell influx (24). However, cell-specific ablation of *Cav1* in retinal Müller glia and neurons (but not vasculature, astrocytes or microglia) suppressed both TLR4-induced retinal inflammatory cytokine/chemokine responses and immune cell infiltration (37). Mechanistic insight into how cell-specific contexts control CAV1 functions and mediate paradoxical inflammatory responses is critical to evaluate the utility of therapeutic targeting of CAV1 (30, 38, 39).

Because most cell types co-express caveolins and cavins, CAV1 functions outside of caveolae have received less attention (40, 41). One notable exception are studies in PC3 prostate cancer cells which stably express CAV1 outside of caveolae due to lack of CAVIN1/PTRF expression (42, 43, 44). In the retina, CAV1 is highly expressed in Müller glia (21, 45, 46, 47, 48, 49) where its expression coincides with Müller glia differentiation (21, 45, 49). While CAV1 is abundantly expressed in Müller glia, the presence of caveolae has not been reported but the CAV1 protein is found predominantly in high molecular weight complexes that are resistant to heat denaturation on reducing SDS-PAGE gels (50). Thus, we hypothesized that CAV1 in Müller glia exists predominantly outside of caveolae and that this non-caveolar localization provides important neuroinflammatory/neuroprotective functional properties. The data presented in this report indicate that Müller glia abundantly express CAV1 with very little CAVIN1/PTRF and are devoid of morphologically identifiable caveolae. Furthermore, non-caveolar CAV1 in Müller glia promotes LPS-induced secretion of pro-inflammatory cytokines, which is suppressed by CAV1 silencing or by sequestration into caveolae by ectopic expression of CAVIN1/PTRF.

## Results

### CAVIN1/PTRF expression in the retina is largely restricted to the vasculature

**Figure 1:**
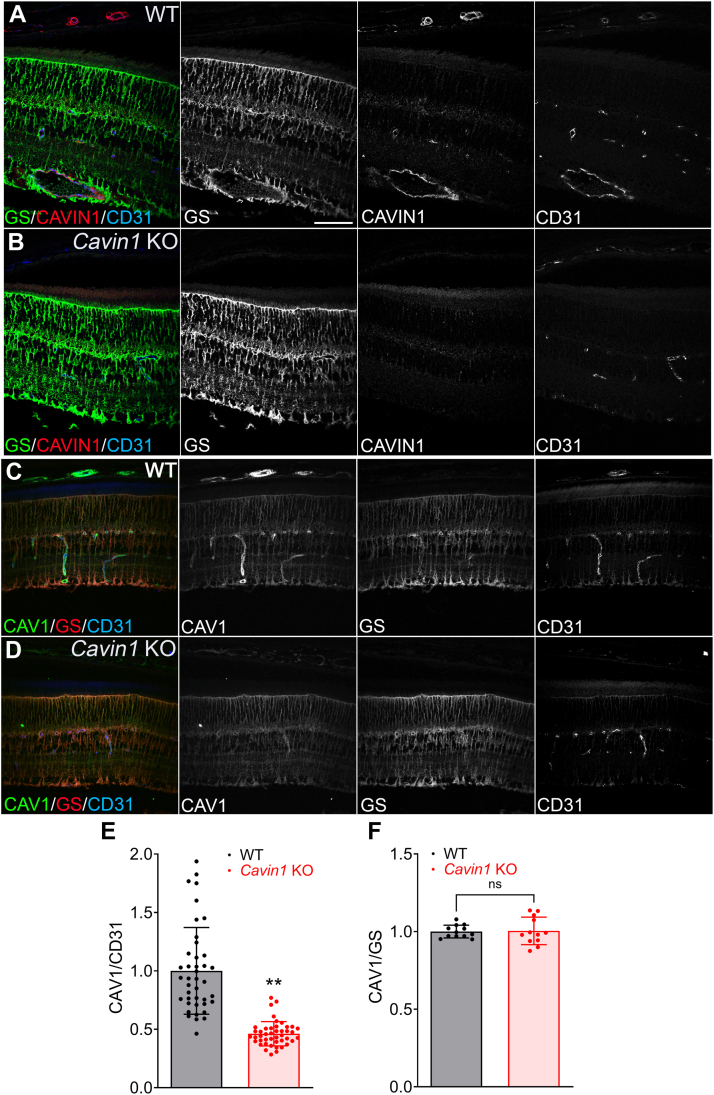
**Müller glia stably express CAV1 in the absence of CAVIN1/PTRF**. *A*, confocal images of a WT retina show immunolocalization of CAVIN1/PTRF in retinal and choroidal blood vessels where it co-localizes with the vascular endothelium marker, CD31. CAVIN1/PTRF was only weakly detectable in the neuroretinal compartment and did not co-localize with glutamine synthetase (GS). *B*, Confocal images of global *Cavin1/Ptrf* KO retina show CAVIN1/PTRF ablation and antibody specificity. *C*, confocal images of WT retina show CAV1 co-localization with CD31 in choroidal and retinal vasculature and with GS to Müller glia. *D*, confocal images of global *Cavin1/Ptrf* KO retina show that CAVIN1/PTRF ablation does not reduce CAV1 expression in Müller glia but results in a dramatic reduction in CAV1 immunoreactivity in retinal and choroidal vasculature. Scale bar = 50 μM. *E–F*, quantitative analysis of immunofluorescence in WT and *Cavin1/Ptrf* KO (n = 3) revealed a significant reduction of CAV1 in retinal and choroidal vascular compartments (CAV1/CD31 ratio) but not Müller glia (CAV1/GS ratio; ∗∗*p* < 0.05; unpaired *t* test). Each data point represents CAV1/CD31 or CAV1/GS in individual ROIs. For statistical analysis, individual ROI data from each mouse were averaged to provide n = 3.

While the expression and cellular localization of CAV1 in the retina is well-established (37, 47, 51), the localization of CAVIN1/PTRF expression has not been established. To evaluate the cell type-specific expression of CAVIN1/PTRF in the retina, we analyzed published single-cell RNAseq datasets from both mouse and human retinas (Fig. S1) (48, 52, 53, 54, 55, 56, 57). While CAV1 is abundantly expressed in Müller glia and vascular cells (both endothelium and mural cells), CAVIN1/PTRF is highly expressed in vascular cells but not Müller glia (Fig. S1). To confirm these transcriptional results, we determined the localization of CAVIN1/PTRF in retinal sections by immunohistochemistry (IHC) and confocal microscopy (Fig. 1). CAVIN1/PTRF co-localized predominantly with the vascular endothelial marker, CD31, in retinal and choroidal blood vessels in WT retinas but did not co-localize with the Müller glial marker, glutamine synthetase (GS) (Figs. 1*A* and S2*A*). Antibody specificity was confirmed by absence of immunostaining in *Cavin1/Ptrf* KO vessels (Fig. 1*B*). Ablation of *Cav1* reportedly downregulates CAVIN1/PTRF expression in almost all tissues and cells studied (10, 13). To confirm that CAVIN1/PTRF is predominantly expressed in the vasculature and not in the neuroretina, we performed IHC on endothelial-specific *Cav1* KO (*endo-Cav1-KO*) (36, 58) and retina-specific *Cav1* KO (*ret-Cav1-KO*) (37) retina sections. Deletion of *Cav1* from the endothelium resulted in significant downregulation of CAVIN1/PTRF expression in retinal and choroidal vessels (Fig. S2*B*). Residual vascular staining in the superficial layer of the retina (yellow arrows in Fig. S2*B*) and the choroid is likely CAVIN1/PTRF localized to perivascular smooth muscle cells as scRNAseq studies show that perivascular cells of the retina express CAVIN1/PTRF (Fig. S1). The observed CAVIN1/PTRF immunolocalization was unaffected by neuroretinal deletion of the *Cav1* gene (Fig. S2*C*). These IHC results were further confirmed by western blots showing reduced CAVIN1/PTRF expression from *endo-Cav1-KO* but not from *ret-Cav1-KO* retinal lysates (Fig. S2, *D–G*).

### Müller glia stably express CAV1 independent of CAVIN1/PTRF

In most cells and tissues, CAV1 protein is stabilized within caveolae by CAVIN1/PTRF, and genetic ablation of *Cavin1/PTRF* results in downregulation of CAV1 (12, 13). As our results indicate that CAVIN1/PTRF is not abundantly expressed in Müller glia while CAV1 is, we reasoned that genetic ablation of *Cavin1/Ptrf* would not perturb Müller glia CAV1. To test this hypothesis, we evaluated CAV1 expression/localization in WT and global *Cavin1/PTRF* KO retina sections by IHC. In WT retinas, CAV1 protein is localized to retinal and choroidal vasculature where it co-localizes with CD31 and in Müller glia where it co-localizes with GS (Fig. 1*C*) as previously reported (37, 45, 59). Consistent with the previously established co-dependence of CAV1 and CAVIN1/PTRF expression in most tissues (12, 13), ablation of *Cavin1/Ptrf* resulted in dramatic loss of CAV1 protein expression in retinal and choroidal vasculature (Fig. 1, *D* and *E*). However, *Cavin1/Ptrf* deletion did not alter CAV1 immunoreactivity in Müller glia (Fig. 1, *D* and *F*). These results indicate that CAV1 stability in Müller glia is not dependent on CAVIN1/PTRF expression.

In most cell types, co-expression of CAVIN1/PTRF and CAV1 is required to form caveolae and CAVIN1/PTRF deficiency results in loss of caveolae (12). Since Müller glia express CAV1 at much higher levels than CAVIN1/PTRF, we reasoned that they would form few, if any, morphologically identifiable caveolae. To test this, we used Fiji (60) to quantify caveolae numbers across vascular endothelial, pericyte, and Müller glial cell membranes from TEM images of mouse retinas (17 individual images from n = 4 mice). Caveolae were abundant on the abluminal side of the retinal vascular endothelium (2.3 ± 1.2 caveolae/μm, mean ± SD, across 134 μm of total endothelial membrane analyzed) and pericytes (0.92 ± 0.59 caveolae/μm, mean ± SD, across 116 μm of total pericyte membrane analyzed) (Fig. S3), as previously reported (20, 61, 62). However, in our extensive survey, we only found 3 morphologically-identifiable caveolae in Müller glia (0.02 ± 0.05 caveolae/μm, mean ± SD, across 159 μm of total Müller glia membrane analyzed). An example of a single caveola is highlighted with a red arrow on the inner limiting membrane at the vitreoretinal interface in Figure S3. In this representative image, numerous caveolae are identified in the endothelium (blue arrows) and a pericyte (orange arrow). Collectively, these results provide compelling evidence that Müller glia stably express CAV1 in the absence of abundant CAVIN1/PTRF and generally lack caveolae implying that CAV1 predominates in non-caveolar domains.

### MIO-M1 Müller glial cells stably express CAV1 without abundant CAVIN1/PTRF

**Figure 2:**
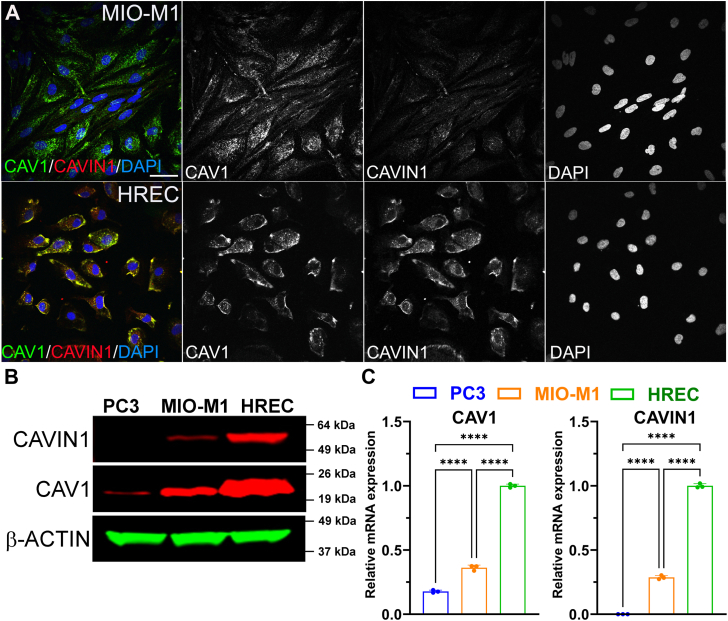
**MIO-M1 Müller glial cells stably express CAV1 without abundant CAVIN1/PTRF**. *A*, immunofluorescence staining of MIO-M1 Müller glia and primary human retinal endothelial cells (HREC) reveals similar CAV1 immunoreactivity between MIO-M1 Müller glia and HREC. CAVIN1/PTRF is virtually undetectable in MIO-M1 Müller glia but is abundant in HREC. Scale bar = 50 μm. *B*, representative Western blots (20 μg protein load) showing CAVIN1, CAV1, and β-actin in MIO-M1 Müller glia, PC3 prostate cancer cells, and HREC. CAVIN1/PTRF protein is reduced in MIO-M1 Müller glia compared to abundant expression in HREC while it is absent from PC3 prostate cancer cells in which the CAVIN1 gene is deleted. CAV1 protein is also reduced in MIO-M1 cells but to a lesser extent than CAVIN1. *C*, transcript expression for CAV1 and CAVIN1/PTRF, as measured by qRT-PCR, mirrors that of protein levels (datapoints represent RNA preparations from n = 3 independent dishes for each cell type), One-way ANOVA with Tukey’s multiple comparison test, ∗∗∗∗*p* < 0.001).

The spontaneously immortalized Müller glial cell line (MIO-M1) is an established model for *in vitro* study of Müller glia (63). To authenticate MIO-M1 Müller glia under our culture conditions, we established that they express known Müller cell markers, GS, vimentin (Vim) and glial fibrillary acid protein (GFAP) (Fig. S4). We compared protein and transcript levels of CAV1 and CAVIN1/PTRF in MIO-M1 Müller glial cells to primary HRECs, which express both proteins and form abundant caveolae, and to the PC3 prostate cancer cell line which expresses non-caveolar CAV1 without CAVIN1/PTRF (10, 15, 43). By immunofluorescence microscopy (Fig. 2*A*), Western blotting (Fig. 2*B*), and qRT-PCR (Fig. 2*C*), MIO-M1 Müller glia expressed significantly lower levels of CAVIN1/PTRF compared to HRECs but higher levels than PC3 cells in which the *CAVIN1/PTRF* gene is deleted (64, 65). Densitometric analysis of near-IR labeled Western blots revealed the ratios of CAVIN/PTRF:CAV1 was 0.31 ± 0.14 in HREC vs 0.14 ± 0.03 in MIO-M1 cells (mean ± SD; n = 3 replicates). Thus, in addition to the CAV1 protein levels being reduced in MIO-M1 cells, the ratio of CAVIN1/PTRF:CAV1 was also reduced compared to HRECs.

### EZH2 histone methyltransferase suppresses Cavin1/PTRF expression in MIO-M1 Müller glia

**Figure 3:**
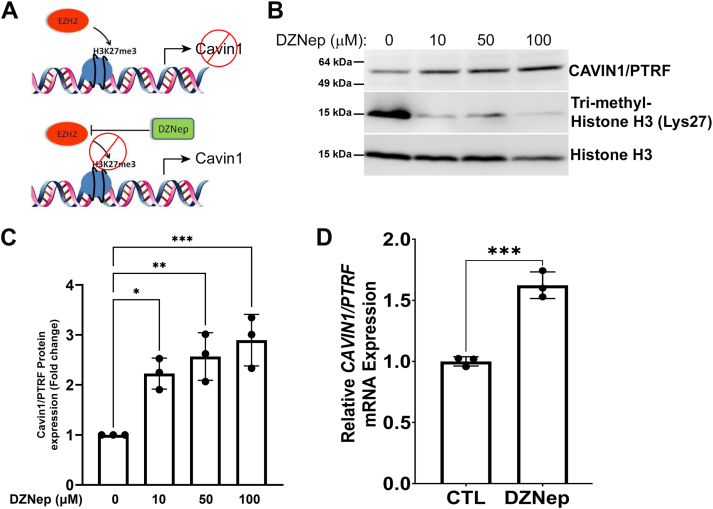
**Inhibition of EZH2 histone methyltransferase induces CAVIN1/PTRF expression in MIO-M1 Müller glia**. *A*, illustration showing putative repression of CAVIN1/PTRF expression by histone trimethylation and relief of repression by inhibition of the EZH2 histone methyltransferase by three-Deazaneplanocin A hydrochloride (DZNep). *B*, representative Western blots and (*C*) densitometric analysis showing induction of CAVIN1/PTRF protein in MIO-M1 Müller glia treated with DZNep and concentrations that reduce histone H3 trimethylation (∗*p* < 0.05, ∗∗*p* < 0.005, ∗∗∗*p* < 0.0005, one-way ANOVA with Dunnett’s multiple comparison tests, datapoints represent protein lysates from n = 3 independent culture dishes for each treatment). *D*, RT-PCR analysis showing induction of CAVIN1/PTRF mRNA in MIO-M1 Müller glia 48 h after 100 μM DZNep treatment (∗∗*p* < 0.05, Welch’s *t* test, datapoints represent RNA preparations from n = 3 independent culture dishes for each treatment).

Unlike PC3 prostate cancer cells in which the *CAVIN1/PTRF* gene is deleted, Müller glia can rapidly upregulate CAVIN1/PTRF mRNA expression when activated by stress (*e.g.*, NMDA toxicity) *in vivo* (Fig. S5, plotted from (52)). We therefore hypothesized that the *CAVIN1/PTRF* gene in Müller glia is silenced under basal conditions by repressive histone methylation, providing more labile repression than DNA methylation which confers more stable gene silencing (66). To test this, we treated MIO-M1 Müller glia with three-Deazaneplanocin A hydrochloride (DZNep), an Enhancer of Zeste two (EZH2) histone methyltransferase-specific inhibitor (Fig. 3*A*). EZH2 is the catalytic subunit of the Polycomb Repressive Complex (PRC2), which methylates Histone (H3) on lysine - 27 for gene repression (67). Treatment of MIO-M1 cells with different DZNep concentrations reduced H3 trimethylation and increased CAVIN1/PTRF protein expression in a dose dependent manner (Fig. 3, *B* and *C*). Similarly, a single dose of 100 μM DZNep markedly increased CAVIN1/PTRF transcript levels in MIO-M1 Müller glia (Fig. 3*D*). As expected, DZNep was unable to induce any detectable expression of CAVIN1/PTRF mRNA in PC3 cells due to deletion of the gene (Fig. S6*A*). To our surprise, 100 μM DZNep treatment of HRECs significantly increased CAVIN1/PTRF mRNA expression although the impact on the already abundant protein expression was more modest (Fig. S6, *B* and *C*). These data suggest EZH2 histone methyltransferase is at least partly responsible for suppression of Müller glial CAVIN1/PTRF gene expression; however, other mechanisms of gene regulation cannot be ruled out. For example, EGR1, a transcription factor involved in ocular growth and expressed in Müller glia (68, 69), can repress CAVIN1/PTRF and CAV1 transcription and regulate of caveolae biogenesis (70).

### CAVIN1/PTRF expression in MIO-M1 Müller glia sequesters non-caveolar CAV1 in caveolae

**Figure 4:**
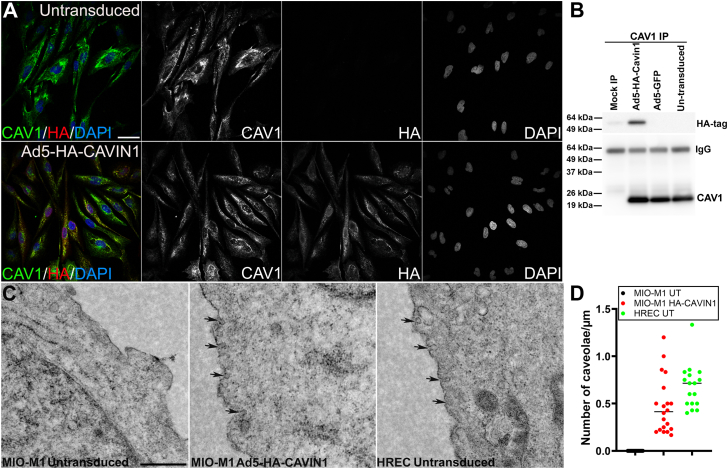
**Adenovirus-delivered CAVIN1/PTRF protein is functional in transduced Müller glia**. *A*, immunocytochemistry showing that expressed HA-tagged CAVIN1/PTRF protein co-localizes with endogenous CAV1 in transduced MIO-M1 Müller glia. Scale bar = 50 μm. *B*, CAV1 immunoprecipitation showing that expressed HA-tagged CAVIN1/PTRF interacts with endogenous CAV1 protein in transduced MIO-M1 Müller glia. *C*, representative electron micrographs showing caveolae (*arrows*) in CAVIN1/PTRF-transduced MIO-M1 Müller glia. Caveolae are absent from untransduced MIO-M1 Müller glia but are present in untransduced HREC which endogenously express CAVIN1/PTRF. Caveolae were identified by their characteristic size and morphology on the membrane. Scale bar = 500 nm. *D*, quantification of caveolae numbers in MIO-M1 Müller glia and HREC. No caveolae were detected in untransduced MIO-M1 Müller glia but they were numerous following CAVIN1/PTRF transduction in numbers similar to those observed in HREC. Caveolae were counted in 19, 20, and 17 non-overlapping images from untransduced (UT) MIO-M1 cells, HA-CAVIN1-transduced MIO-M1 cells, or UT HREC, respectively, and reported per μm of membrane. UT, untransduced.

After establishing that the MIO-M1 Müller glia can model endogenous Müller glia, we constructed an Adenovirus Type five delivery system, to express hemagglutinin (HA)-tagged CAVIN1/PTRF in MIO-M1 Müller glia (Figs. S4 and S7) to sequester CAV1 and form caveolae as co-expression of CAVIN1/PTRF and CAV1 is required to form caveolae (12). Upon transduction with HA-tagged CAVIN1/PTRF adenoviral particles, MIO-M1 Müller glia expressed the predicted molecular weight protein that was recognized by both HA and CAVIN1/PTRF antibodies (Fig. S7, *B* and *C*). Expression of CAVIN1/PTRF did not affect CAV1 protein levels (Fig. S4) and did not dramatically alter its subcellular localization (Fig. 4*A*) although the resolution of confocal microscopy is not sufficient to evaluate subtle changes in localization between caveolae and non-caveolar domains. To confirm that the HA-tagged CAVIN1/PTRF protein was functional, we performed immunostaining and immunoprecipitation (IP) using CAV1 primary antibodies. Our results showed that HA-tagged CAVIN1/PTRF co-localized and interacted with endogenous CAV1 (Fig. 4, *A* and *B*). To determine if HA-CAVIN1/PTRF can drive formation of caveolae, we evaluated untransduced (n = 19) and transduced (n = 20) MIO-M1 cells and untransduced HRECs (n = 17) by TEM analysis of non-overlapping images. Caveolae were not identified in native, untransduced MIO-M1 cells (Fig. 4, *C* and *D*). Transduction with HA-tagged CAVIN1/PTRF resulted in formation of morphologically identifiable caveolae in numbers similar to those observed in HRECs (0.47 ± 0.29 vs 0.68 ± 0.23 caveolae/μm; mean ± SD; Fig. 4, *C* and *D*). Taken together, these results indicate that exogenously expressed CAVIN1/PTRF is functional and establish a novel model to evaluate the function of caveolar and non-caveolar CAV1 pools in Müller glia.

### Silencing CAV1 suppresses TLR4-mediated IL - 6 family cytokine production

**Figure 5:**
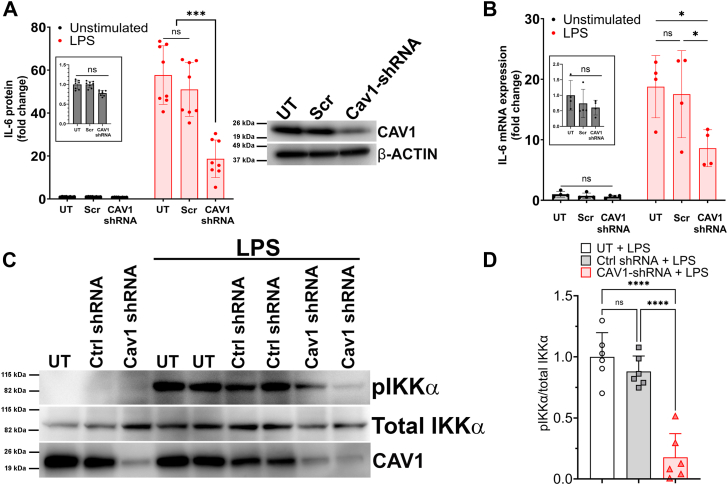
**Silencing non-caveolar CAV1 in MIO-M1 Müller glia suppresses TLR4-mediated IL-6 response.***A*, CAV1 silencing in MIO-M1 Müller glia significantly reduces TLR4-induced IL - 6 protein levels in tissue culture media. Cells were either untransduced (UT) or transduced with Ad5-control (Scr) or Ad5-CAV1-shRNA viruses for 96 h, followed by stimulation with 0.02 μg/ml LPS. After LPS stimulation, IL - 6 levels in tissue culture medium was significantly increased in all treatments compared to unstimulated controls. However, in CAV1-silenced cells, LPS-induced IL - 6 protein was significantly reduced (Two-way ANOVA with Tukey’s multiple comparison tests, ∗∗∗*p* < 0.0005 in CAV1 silenced, LPS-stimulated cells, datapoints represent protein levels from media from individual cell culture dishes). Basal IL - 6 secretion was not significantly different between groups (*inset, A*). Representative Western blots show effective CAV1 silencing. *B*, qPCR analysis of CAV1-silenced MIO-M1 cells. CAV1 silencing reduces IL - 6 mRNA transcript levels in MIO-M1 cells. Cells were transduced and stimulated as in *A*. Datapoints represent RNA preparations from individual cell culture dishes. *C*, representative Western blots and (*D*) semiquantitative densitometric analysis showing significantly blunted IKKα phosphorylation in CAV1-silenced MIO-M1 Müller glia, in response to TLR4 activation by 30 min of LPS stimulation (One-way ANOVA with Tukey’s multiple comparison tests, ∗∗*p* < 0.005, datapoints represent densitometric analysis of bands from six samples from three blots). Cav1, CAV1-shRNA; Scr or Ctrl shRNA, scrambled-shRNA; UT, untransduced.

Our results provide compelling evidence that CAV1 in Müller glia predominantly resides in non-caveolar domains. We previously reported that genetic deletion of CAV1 in the neuroretina (predominantly Müller glia CAV1) but not elsewhere suppresses TLR4-induced retinal inflammatory cytokine production and immune cell infiltration, suggesting that non-caveolar CAV1 promotes innate immune receptor activation in Müller glia (37). As MIO-M1 Müller glia respond to TLR4 stimulation (71), we evaluated whether silencing non-caveolar CAV1 *via* adenoviral transduction of shRNA in MIO-M1 Müller glia similarly blunts TLR4-mediated signaling. As shown in Fig. 5*A*, LPS stimulation induced a more than 50-fold increase in IL - 6 protein in untransduced and Ad5-scrambled shRNA (Scr) control media. Intriguingly, silencing CAV1 resulted in a significant reduction in TLR4-mediated IL - 6 protein secretion into cell culture media but did not significantly impact basal secretion (Fig. 5*A*, *inset*). As CAV1 reportedly regulates retinal neuroprotective signaling (51, 72, 73, 74), we tested whether CAV1 silencing blunts production of the neuroprotective IL - 6 family cytokine, leukemia inhibitory factor (LIF) (75, 76). Indeed, LPS-mediated TLR4 activation in MIO-M1 Müller glia induced LIF secretion, and this response was significantly blunted in cells in which CAV1 was silenced (Fig. S8).

To further elucidate the functional role of non-caveolar CAV1 in modulating inflammatory responses, we tested whether silencing CAV1 reduced IL - 6 mRNA expression, *i.e.*, is the regulation transcriptional or posttranslational? Some studies indicate that CAV1 acts at the level of the TLR4 receptor to modulate downstream signaling and cytokine expression (31, 32, 34, 36, 77) while in cancer cells, CAV1 participates in basal cytokine secretory pathways (15, 43, 78). As shown in Figure 5*B*, CAV1 silencing significantly reduced TLR4-induced IL - 6 mRNA expression that mirrors the impact on IL - 6 protein levels in medium (Fig. 5*A*) suggesting that CAV1 impacts TLR4-mediated IL - 6 transcription. To further support these findings, we interrogated the TLR4-induced NF-κB signaling pathway. Under basal conditions, inactive NF-κB is sequestered in the cytosol where it is bound to the IκBα inhibitor. Upon innate immune receptor activation (*e.g.*, LPS-induced TLR4 activation), IκB Kinase (IKK) is phosphorylated and activated to drive phosphorylation of IκBα, resulting in dissociation from the NF-κB complex and translocation of the NF-κB p65 and p50 subunits into the nucleus where they drive transcription of pro-inflammatory cytokines like IL - 6 (79). LPS stimulation of Müller glia induced the time-dependent phosphorylation of IKKα (Fig. S9*A*) and induced NF-κB/p65 nuclear localization (Fig. S9, *B*–*D*). Upon CAV1 silencing there was significantly reduced LPS-induced IKK phosphorylation (Fig. 5, *C* and *D*) and nuclear localization of NF-κB/p65 (Fig. S9, *E* and *F*). Additionally, CAV1 silencing significantly impacted intracellular IL - 6 protein levels after LPS stimulation in MIO-M1 Müller glial cell lysates (Fig. S10). Collectively, these results indicate that non-caveolar CAV1 promotes TLR4/NF-κB signaling, IL - 6 transcription and secretion but not basal secretion in MIO-M1 Müller glia.

### Sequestering non-caveolar CAV1 in caveolae by expressing CAVIN1/PTRF suppresses TLR4 signaling and IL - 6 release

**Figure 6:**
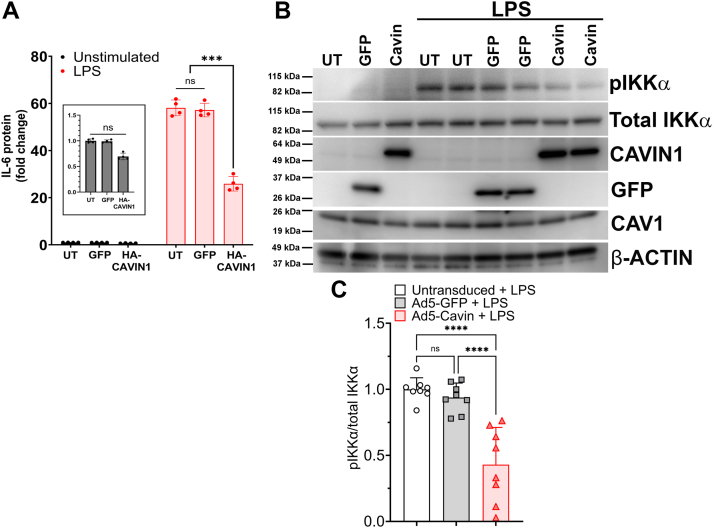
**CAVIN1/PTRF expression in MIO-M1 Müller glia suppresses TLR4-mediated IL-6 response.***A*, MIO-M1 Müller glia were transduced with Ad5 virus (either HA-tagged CAVIN1/PTRF or GFP) or left untransduced. Cells were stimulated with 0.02 μg/ml LPS for 6 h and cell culture media was collected for IL - 6 quantification by ELISA. After LPS stimulation, IL - 6 levels in tissue culture medium was significantly increased in all treatments compared to unstimulated controls. However, in HA-CAVIN1 transduced cells, LPS-induced IL - 6 protein was significantly reduced (Two-way ANOVA with Tukey’s multiple comparison tests, ∗∗∗*p* < 0.0005 in HA-CAVIN1 transduced, LPS-stimulated cells, datapoints represent protein levels from media from individual cell culture dishes). Basal IL - 6 secretion was not significantly different between groups (*inset, A*). *B*, representative Western blots show IKKα phosphorylation, CAVIN1/PTRF and control transductions, and CAV1 and β-actin levels. *C*, semiquantitative densitometric analysis showing significantly blunted IKKα phosphorylation in CAVIN1/PTRF-transduced MIO-M1 Müller glia in response to TLR4 activation. Cells were stimulated with 0.5 μg/ml LPS for 30 min (One-way ANOVA with Tukey’s Multiple comparison tests, ∗*p* < 0.05, datapoints represent densitometric analysis of bands from eight samples from four blots). GFP, Ad5-GFP control; HA-Cavin1, Ad5-HA-CAVIN1; UT, untransduced.

After establishing that that *in situ* Müller glia and the MIO-M1 Müller glia cell line predominantly express CAV1 outside of caveolae and that silencing CAV1 suppresses inflammatory cytokine production, we tested whether the non-caveolar localization of CAV1 is critical to support inflammatory signaling. We hypothesized that sequestering CAV1 within caveolae by expressing CAVIN1/PTRF would blunt TLR4-mediated responses. To test this, we expressed HA-tagged CAVIN1/PTRF in Müller glia to sequester CAV1 into caveolae (as established in Fig. 4) and measured IL - 6 levels in tissue culture media in unstimulated cells and those in which TLR4 was activated by LPS. As shown in Fig. 6*A*, LPS stimulation significantly increased IL - 6 levels in tissue culture media. Basal IL - 6 secretion (unstimulated; inset in Fig. 6*A*) was slightly reduced in HA-CAVIN1/PTRF-transduced cells but this did not reach statistical significance. However, in LPS-stimulated cells, expression of CAVIN1/PTRF significantly suppressed TLR4-mediated IL - 6 release (Fig. 6*A*). Similar to CAV1 silencing, adenoviral expression of CAVIN1/PTRF significantly reduced LPS-induced IKK phosphorylation (Fig. 6, *B* and *C*). Thus, sequestration of CAV1 into caveolae by expressing CAVIN1/PTRF blunts LPS-induced innate immune signaling.

### Silencing CAV1 in HRECs promotes TLR4-mediated IL - 6 release

**Figure 7:**
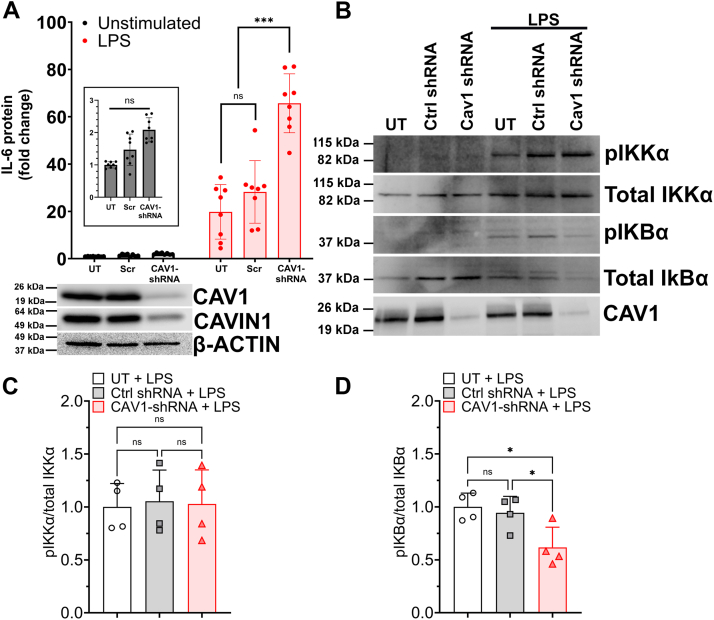
**CAV1 silencing in HREC enhances TLR4-mediated IL-6 response to LPS.***A (upper panel)*, similar to Müller glia, activation of TLR4 in HREC significantly increased IL - 6 protein levels in all LPS-stimulated groups. However, unlike Müller glia, silencing CAV1 in HREC significantly *enhanced* IL - 6 secretion in LPS-treated groups (Two-way ANOVA with Tukey’s multiple comparison tests, ∗∗∗*p* < 0.0005 in CAV1 silenced, LPS-stimulated HREC compared to both UT and Scr controls, datapoints represent protein levels from media from individual cell culture dishes). Silencing CAV1 in unstimulated HREC modestly increased basal IL - 6 secretion but this did not reach statistical significance (*inset, A*). Representative Western blots showing efficient silencing of CAV1 expression and concomitant downregulation of CAVIN1/PTRF in CAV1 silenced HREC (*lower panel*, *A*). *B*, representative Western blots showing phosphorylation of IKKα and decreased phosphorylated IκBα in CAV1 silenced HREC. Semiquantitative densitometric analysis showed no significant increase in phosphorylated IKK (*C*) but a significant decrease in phosphorylated IκBα (*D*) in CAV1-silenced HREC (One-way ANOVA with Tukey’s multiple comparison tests, ∗*p* < 0.05, datapoints represent densitometric analysis of bands from four samples from four blots).

We previously reported the puzzling finding that global ablation of *Cav1*, *in vivo*, resulted in a significant suppression of pro-inflammatory cytokine levels in retinal lysates while paradoxically increasing infiltration of immune cells and leukostasis in the retinal vasculature (24). In this early paper, we speculated that CAV1-mediated inflammatory regulation could be cell-context specific, that is, that global *Cav1* deletion might suppress pro-inflammatory cytokines/chemokines in Müller glia but might, at the same time, increase inflammatory responses in the retinal vascular endothelium. In many studies outside of the eye, ablation of *Cav1* is pro-inflammatory (80, 81). In fact, we previously observed that endothelium-specific deletion of *Cav1* increases LPS-induced inflammation in the lung (36) in contrast to the decreased inflammatory response observed from retina-specific *Cav1* ablation (37). Given that CAV1 protein is predominantly non-caveolar in Müller glia but is localized to caveolae in endothelial cells, we hypothesized that CAV1 in these contexts might differentially regulate inflammatory signaling. To evaluate the impact of caveolar CAV1 on inflammatory responses, we silenced CAV1 expression in HREC cells and stimulated them with LPS. Ad5-CAV1-shRNA not only reduced CAV1 but also CAVIN1/PTRF protein levels in HRECs (Western blot in Fig. 7*A*) suggesting a reduction in caveolae. In contrast to Müller glia, silencing CAV1 in HRECs significantly enhanced LPS-induced IL - 6 production (Fig. 7*A*). To assess the impact of CAV1 silencing on TLR4/NF-κB signaling, we evaluated phosphorylation of IKKα and Iκβ. As indicated in Figure 7, *B* and *C*, LPS-induced phosphorylation of IKKα was slightly but not significantly increased in CAV1-silenced HRECs (unlike the suppressed phosphorylation in Müller glia). Consistent with the idea that NF-κB signaling is increased in CAV1-silenced HREC, we observed a significant decrease in total Iκβ (as well as phospho-Iκβ) suggesting degradation of the regulatory NF-κB subunit (Fig. 7, *B* and *D*). These results are opposite to the response observed in Müller glia, supporting the idea that CAV1-mediated inflammatory control is cell context-specific. As CAV1 is predominantly localized to caveolae in the vascular endothelium and is non-caveolar in Müller glia, our results are consistent with the idea that caveolar localization differentially impacts CAV1-mediated inflammatory regulation.

## Discussion

Although discovered in the 1950s by George Palade (82, 83), the role of caveolae as critical modulators of ocular disease has only recently received attention. CAV1, the signature protein of caveolae, was the first protein found to be essential and necessary for caveolae biogenesis (3, 84, 85). This led to the initial belief that CAV1 was the sole protein required for caveolae formation, since CAV1 deletion significantly inhibits caveolae biogenesis (85, 86, 87). Subsequently, CAVIN1/PTRF was also shown to be required for caveolae formation as CAVIN1/PTRF deficiency also results in loss of caveolae (10, 12). In most cellular contexts, CAV1 and CAVIN1/PTRF are co-expressed and loss of 1 results in loss of the other (13, 88, 89, 90). In contrast to this general principle, we report here that CAV1 is highly expressed in retinal Müller glia in the absence of CAVIN1/PTRF and preferentially resides outside of caveolae. Based on the analysis of several previously published scRNA-seq datasets from both mouse and human retinas (Fig. S1) and from quantification of caveolae in mouse retinal cells, *in situ* (Fig. S3), our data suggest that the majority of Müller glial cells (at least quiescent ones) are deficient in both CAVIN1/PTRF expression and in caveolae when compared to cells that abundantly contain caveolae (*e.g.*, vascular endothelium). However, the analysis of scRNA-seq data suggest that a subset of Müller glia may express higher levels of CAVIN1/PTRF (see datasets from Voigt *et al.*, in lower panels of Fig. S1), but these numbers are small. Furthermore, data presented in Figure S5 suggests that upon retinal injury, (*e.g.*, NMDA toxicity) that some Müller glia may upregulate CAVIN1/PTRF expression suggesting the possibility of redistribution of at least some CAV1 to caveolae in response to injury. However, the significance of this upregulation merits further investigation.

Our results are in agreement with published transcriptomic data showing CAV1 but not CAVIN1/PTRF to be enriched in Müller glia (47, 48) with CAVIN1/PTRF instead highly expressed in retinal vascular cells (48, 52). PC3 prostate cancer cells, which, like Müller glia, are deficient in CAVIN1/PTRF expression, represent the best-described cell type in which CAV1 predominantly resides outside of caveolae (10, 43, 44). However, unlike murine and human Müller glia, PC3 cells lack CAVIN1/PTRF because of a homozygous deletion in chromosome 17 where the *CAVIN1/PTRF* gene resides (64, 65). By contrast, CAVIN1/PTRF expression in murine Müller glia can be induced transiently in response to stress *in vivo* (Fig. S5), suggesting that expression is actively repressed under basal conditions. Our results suggest that one repressive mechanism is *via* histone methylation (Fig. 3) but further analysis of CAVIN1/PTRF gene regulation in Müller glia is warranted in future studies. In addition, our results establish clearly that CAV1 is stably expressed without significant CAVIN1/PTRF and that global deletion of the *Cavin1/Ptrf* gene does not affect CAV1 protein expression in Müller glia (although it does dramatically reduce expression in retinal and choroidal vasculature). Thus, the mechanism by which CAV1 is stabilized in the absence of CAVIN1/PTRF also merits further study.

There is compelling evidence linking CAV1 expression to regulation of innate immune responses. Previously published data suggest an anti-inflammatory role for CAV1 in extra-ocular tissue contexts (80, 81). For example, global *CAV1* ablation significantly enhanced systemic inflammatory response to LPS, characterized by increased pro-inflammatory cytokine secretion (80). *Cav1* ablation also increased lethality in a mouse model of sepsis (81). However, work in our lab suggests a pro-inflammatory role for CAV1 in the retina. In our neuroretina-specific *Cav1* KO model, we observed suppression of LPS-induced pro-inflammatory cytokine secretion and immune cell influx into the retina (37). A fundamental question is why the immunomodulatory functions of CAV1 differ between the retina and some other cell types. We hypothesize that the unique non-caveolar localization of CAV1 in Müller glia explains this paradox. Our *in vitro* data in MIO-M1 Müller glia support the hypothesis that non-caveolar CAV1 regulates retinal innate immune responses by regulating secretion of pro-inflammatory cytokines. As seen in Figure 2 and 4*C*, MIO-M1 Müller glia can model endogenous Müller glia, since CAV1 in these cells exists preferentially outside of caveolae. Expression of HA-tagged CAVIN1/PTRF in MIO-M1 Müller glia resulted in significantly higher numbers of caveolae. We also show that silencing CAV1 or sequestering it into caveolae by expressing CAVIN1/PTRF results in significantly suppressed TLR4-mediated responses in Müller glia but surprisingly enhanced responses when CAV1 is silenced in vascular endothelial cells.

CAV1 can reportedly regulate TLR4 signaling by two mechanisms: (1) direct interaction resulting in either stimulation or inhibition of the receptor (91); and (2) by an indirect mechanism involving over-activation of endothelial nitric oxide synthase (eNOS), resulting in NF-ƙB activation (92). We have observed that when *Cav1* is ablated globally in mice, TLR4-induced activation inflammatory cytokine release is suppressed, while immune cell influx into the retina is paradoxically enhanced (24). This seemingly contradictory immune regulatory role for CAV1 may be cell-context dependent. Unlike in Müller glia, CAV1 in retinal endothelial cells resides predominantly within caveolae, as they co-express CAV1 and CAVIN1/PTRF. CAV1 silencing in HRECs significantly downregulated CAVIN1/PTRF expression (Fig. 7*A*), in agreement with dogma that CAV1 and CAVIN1/PTRF expression are coupled (10, 12, 13). Intriguingly, IL - 6 response to LPS in HRECs was opposite to what we observed in MIO-M1 Müller glia (Fig. 7). This observation raises a fundamental question as to why the same cell manipulation (*i.e.*, CAV1 silencing) produces opposing effects in different cell types. Previously, it has been reported that endothelial CAV1 deletion enhances TLR4 signaling in the lung (36), but neuroretinal deletion suppresses it (37). Furthermore, ectopic expression of CAVIN1/PTRF in PC3 cells sequesters non-caveolar CAV1 into caveolae, resulting in suppression of IL - 6 secretion (43). These results suggest that caveolae suppress TLR4 signaling but that non-caveolar CAV1 enhances such responses. These findings may have broad implications as caveolae are known to disassemble or flatten in response to mechanical stimulation resulting in a transient state in which CAV1 is non-caveolar (93). Under such conditions, our results would imply that the transition to non-caveolar CAV1 domains could transiently promote TLR4 responses. Intriguingly, in chondrocytes mechanical stimulation can activate TLR4 to induce IL - 6, and this is enhanced when CAV1 is silenced (94).

In the retina, IL - 6 family cytokines can serve a neuroprotective role in response to a variety of injurious stimuli including light, intraocular pressure, retinal detachment, and excitotoxicity (95, 96, 97, 98). Endogenous IL - 6 slows photoreceptor death during periods of separation from retinal pigment epithelium (97), and has established roles in protecting retinal ganglion cells (RGC) from pressure-induced death (98). This effect is similar to the neuroprotective effect of ciliary neurotropic factor (CNTF) and leukemia inhibitory factor (LIF), IL - 6 family cytokines known to protect against retinal degeneration (75, 76, 99). IL - 6 promotes neuroprotection through IL - 6 receptor (IL-6R)-mediated activation of STAT3 (97). Our data indicate that IL - 6 responses are regulated by CAV1, both in our neuroretina-specific *Cav1* KO model, *in vivo* (37) and in Müller glia *in vitro*. Similar to the effect on IL - 6, silencing non-caveolar CAV1 in Müller glia also suppresses LIF production (Fig. S8). Thus, we speculate that the predominant non-caveolar localization of CAV1 in Müller glia may serve to promote neuroprotective cytokine production and may explain why CAV1 is predominantly non-caveolar in these cells.

In conclusion, we demonstrate that Müller glial CAV1 exists predominantly in non-caveolar domains and promotes pro-inflammatory signaling that is attenuated by either CAV1 silencing or sequestration into caveolae. This cell context-specific function contrasts with endothelial CAV1, where caveolar localization suppresses inflammation. Because the functional properties of Müller glia are well-known, they represent a unique model to manipulate and study the structure and functions of CAV1 in and outside of caveolae. Understanding the mechanisms by which non-caveolar CAV1 in Müller glia regulates retinal inflammatory responses will be vital in developing new and improved therapies against chronic ocular inflammation.

## Experimental procedures

### Animals

Eyes from *Cavin1/Ptrf* KO mice generated as described in Liu *et al.* 2008 (12) were generously provided by Dr Paul Pilch. Retina-specific *Cav1* KO (*ret-Cav1 KO*) mice were previously generated (37) by crossing mice hemizygous for Chx10-cre (Tg(Chx10-EGFP/cre-ALPP)2Clc; stock no. 005105; The Jackson Laboratory) with *Cav1* floxed mice carrying loxP sites flanking exon two of the *Cav1* gene (100). Endothelium-specific *Cav1* KO (*endo-Cav1 KO*) mice were previously generated by crossing *Cav1* floxed mice with hemizygous Tie2-Cre (B6.Cg-Tg(Tek-cre)1Ywa/J; stock number 008863, The Jackson Laboratory) as described (36, 58).

All animal experiments complied with the Association of Research in Vision and Ophthalmology Statement for the Use of Animals in Ophthalmic and Vision Research and were approved by the Institutional Animal Care and Use Committee at the University of Oklahoma Health Sciences Center.

### Cell lines and culture conditions

Immortalized Müller glia (MIO-M1) cells at passage 20 were sent to us by G. Astrid Limb, from University College London and were authenticated by STR profiling by ATCC. We used cells from passages 21 to 45 for experiments; Dr Limb has reported similar passage numbers are comparable to primary Müller glia at passage three (63). Further validation under our culture conditions was done Western blot analysis of expression of Müller glial markers (Fig. S4). Primary HRECs were purchased from Cell Systems (Cat#: ACBRI 181). Human embryonic kidney (HEK 293T) cells were purchased from ATCC (Cat#: ATCC CRL - 3216). Prostate cancer (PC3) cells were commercially obtained from ATCC (Cat#: CRL - 1435). MIO-M1 Müller glia and HEK 293T cells were cultured in DMEM (1X) + GlutaMax-I (Invitrogen, Cat#: 10,566-016). PC3 cells were cultured in F-12K medium (ATCC Cat#: 30-2004). MIO-M1 Müller glia and PC3 culture media were supplemented with 10% (v/v) fetal bovine serum (FBS) and 1% penicillin/streptomycin. HREC cells were cultured in Endothelial Cell Basal Medium - 2 (Lonza, Cat# CC - 3156), supplemented with 2% FBS, hydrocortisone, human fibroblast growth factor, vascular endothelial growth factor, insulin-like growth factor - 1, ascorbic acid, human endothelial growth factor and gentamicin-amphotericin B. All cell lines were maintained at 37 °C in a humidified atmosphere of 5% CO_2_. CAVIN1/PTRF protein expression and CAV1 silencing in MIO-M1 Müller glia were achieved by Adenovirus serotype five (Ad5)-mediated delivery. CAV1 silencing was performed using CAV1-shRNA (Ad-GFP-U6-h-Cav1-shRNA (shADV - 204148, Vector BioLabs).

To evaluate the role of histone methylation on CAVIN1/PTRF, MIO-M1 cells, PC3 cells and HRECs were treated with indicated concentrations of the histone methylation inhibitor, DZNep three-Deazaneplanocin A hydrochloride (DZNep; Tocris Bioscience; Cat#: 4703). Protein or mRNA were collected 48 h after treatment to evaluate the effect on CAVIN1/PTRF expression by Western blotting and qRT-PCR, respectively. The reduction in histone H3 methylation was confirmed by Western blotting.

### Adenovirus plasmid construction and virus production

An adenoviral system was generated to deliver HA-tagged CAVIN1/PTRF using the RAPAd CMV Adenoviral Expression System (Cell BioLabs, Cat#: VPK - 252) according to the manufacturer’s instructions. CAV1 silencing was performed using CAV1-shRNA (Ad-GFP-U6-h-Cav1-shRNA (shADV - 204148, Vector BioLabs). For control of silencing, scrambled shRNA with GFP (Ad-GFP-U6-shRNA, Cat#: 1122, Vector Biolabs) was used. Recombinant adenovirus serotype five (Ad5) production was performed as previously described (101, 102). HA-tagged CAVIN1/PTRF plasmids purchased from Addgene (Cat#: 50441) were first amplified by PCR and cloned into pacAd5 CMVK-NpA shuttle vector. Viruses were produced by recombination between the shuttle vector and a novel pacAd5 9.2 to 100 adenoviral backbone vector. Both plasmids were linearized with PacI restriction enzyme and co-transfected into HEK293 T cells for viral packaging. Recombinant viruses were generated within 7 to 14 days in culture, harvested and purified by cesium chloride (CsCl) density gradient centrifugation. For efficient cell transduction with minimal toxicity, we settled on a multiplicity of infection (MOI) of ten. A time-course experiment was performed to determine the optimal time point for CAVIN1/PTRF expression and efficient CAV1 knockdown. MIO-M1 cells were transduced with HA-tagged CAVIN1/PTRF (Ad5-HA-CAVIN1/PTRF) for 48 to 96 h depending on the experiment performed. For example, to determine HA, CAV1 and CAVIN1/PTRF expression, MIO-M1 cells were transduced for 48 h. To measure IL - 6 or LIF response by ELISA, MIO-M1 cells were transduced with CAV1-shRNA and Ad5-HA-CAVIN1 adenoviruses for 96 h.

### Western blotting

Retinas and cells for Western blotting were lysed in buffer containing 120 mM octylglucoside, 150 mM NaCl, 10 mM Tris-HCl pH 7.4, 0.5 mM EDTA, 0.1% Triton X-100 and protease inhibitor cocktail (Roche Cat#: 11836153001). Cells were washed twice with ice-cold PBS and lysed directly on plates, while retinas were homogenized by sonication in lysis buffer. The resulting lysates were kept at constant agitation for 30 min, followed by centrifugation to clear lysates. Proteins were quantified using the BCA assay (ThermoFisher Scientific Cat#: 23227) according to the manufacturer’s instructions using BSA as a standard. Equal amounts of proteins were separated by SDS-PAGE, transferred to nitrocellulose membranes and probed with primary antibodies indicated (Table S1). Secondary antibodies conjugated to horseradish peroxidase (HRP) were used (Table S1). Western blot imaging was performed using either the *In Vivo* F-Pro Imaging system (Carestream Health Inc) or the Alliance Q9 Imaging system (Uvitec, Cambridge, UK) and semiquantitative densitometry using Fiji (60).

To quantitively evaluate protein levels of CAVIN1/PTRF and CAV1 in cultured cells, we imaged Western blots using a Li-Cor Odyssey CLx Imager (Li-Cor Biotechnology). Secondary antibodies to detect CAVIN1/PTRF, CAV1 and β-actin primary antibodies were near-infrared Alexa Fluor conjugates (see Table S1). Linearity of near-IR fluorescence detection was evaluated by serial dilution of cell lysates to adjust protein loads to facilitate cell-type comparisons. Densitometric analysis of Li-Cor blots was done using Li-Cor Bio Image Studio software.

### Immunohistochemistry and immunocytochemistry

Enucleated eyes were fixed in Prefer fixative (Anatech Ltd) and embedded in paraffin prior to sectioning. Cells on coverslips were washed twice with PBS and fixed in 4% paraformaldehyde for 10 min. Retina sections and cells were blocked for one hour in blocking buffer containing 10% normal horse serum and 0.1% Triton X-100 in phosphate-buffered saline (PBS). Retinal sections and cells were incubated overnight with primary antibodies indicated (Table S1). Primary antibodies were detected with appropriate fluorescently labeled secondary antibodies (Table S1). Nuclei were counterstained with DAPI (4,6-diamidino-2-phenylindole; Sigma-Aldrich). Antibody specificity was validated using knockout mice (for CAV1 and CAVIN1/PTRF), shRNA silencing (for CAV1 in cells), and by omission of primary antibodies. Images were captured by an Olympus FV1200 laser scanning confocal microscope using FluoView software (Olympus, Tokyo, Japan).

To evaluate the impact of CAVIN1/PTRF deletion on CAV1 protein levels in Müller glia and retinal vascular endothelium, we co-immunolabeled retinal sections with antibodies against CAV1, CD31 (vascular endothelium), and glutamine synthetase (Müller glia; see Fig. 1). Using Fiji, individual color channels were split into grayscale channels for selection of regions of interest (ROIs). For vascular endothelium, ROIs of retinal vessels (CD31-positive pixels) were selected using the freehand tool on the CD31 channel. These ROIs were then copied to the CAV1 channel of the same image, allowing for intensity measurements to be made in the same ROIs for CAV1 and CD31. ROIs from 41 distinct vessels from three independent WT mice and 44 distinct vessels from three independent Cavin1/PTRF KO mice were evaluated. For Müller glia, rectangular ROIs were selected in the outer nuclear layer (ONL) region using the glutamine synthetase channel. The ONL was chosen as it is avascular and only contains the spanning Müller glia and photoreceptor cells, which express low levels of CAV1. Thus, care was taken to avoid selection of vasculature to evaluate the Müller glial CAV1 expression. For each retina section, three non-overlapping ONL ROIs were selected from the same n = 3 WT and KO sections for intensity measurements. Raw intensities of CD31 and glutamine synthetase were not significantly different between genotypes, so CAV1 intensity in each ROI was normalized to the corresponding intensity of CD31 (for vessels) or glutamine synthetase (for Müller glia) to provide CAV1/CD31 (Fig. 1*E*) or CAV1/glutamine synthetase (Fig. 1*F*) ratios.

### ELISA

The levels of IL - 6 and LIF proteins in cell culture supernatants were determined with ELISA assay kits (Sigma-Aldrich, Cat# RAB0306 and RAB0339, respectively), according to the manufacturer’s instructions. MIO-M1 cells were transduced with either CAV1-shRNA, HA-tagged CAVIN1/PTRF or control virus for 96 h. After transduction, cells were stimulated with or without 0.02 μg/ml LPS (Sigma-Aldrich, Cat# L2262). Cell culture supernatant was collected at indicated time points after LPS challenge to assess IL - 6 levels by ELISA. To measure intracellular IL - 6 levels, cells were lysed in buffer containing 120 mM octylglucoside, 150 mM NaCl, 10 mM Tris-HCl pH 7.4, 0.5 mM EDTA, 0.1% Triton X-100 and protease inhibitor cocktail (Roche Cat#: 11836153001) and the ELISA assay was done according to the manufacturer’s instructions.

### Quantitative RT-PCR

RNA was isolated from cells using RNeasy Mini Kit (Qiagen Cat#: 74104), including a genomic DNA removal step (Qiagen Cat#: 79254), according to manufacturer’s instructions. Briefly, at collection time, cells were washed with ice-cold 1X PBS and immediately lysed in 600 μl buffer RLT supplemented with 2-β mercaptoethanol. The lysate was transferred to a fresh tube, thoroughly mixed with 600 μl of 100% ethanol, and sequentially loaded onto a RNeasy MinElute column. RNA was quantified with a Nanodrop One spectrophotometer (Thermo Fisher Scientific) and its quality assessed by RNA ScreenTape with a 4150 TapeStation analyzer (Agilent Technologies).

Confirmation of gene expression levels was performed with qPCR as described (103). Briefly, cDNA was synthesized with the ABI High-Capacity cDNA Reverse Transcription Kit (Applied Biosystems Inc) from 1 μg of purified RNA. qPCR was performed with gene-specific primer probe fluorogenic exonuclease assays (TaqMan, Life Technologies, Table S2) and the QuantStudio 12K Flex Real-Time PCR System (Applied Biosystems). Relative gene expression (RQ) was calculated with Expression Suite v 1.0.3 software using the 2^−ΔΔCt^ analysis method with HPRT1 as an endogenous control.

For IL - 6 mRNA extraction and quantitative RT-PCR, MIO-M1 cells were collected 6 h after LPS treatment and snap frozen in liquid nitrogen for storage at −80 °C until RNA isolation. Total RNA was isolated using the Quick-RNA Miniprep Plus Kit (Zymo Research, Cat# R1058) and stored in elution buffer at −80 °C until further use. RNA concentration was quantified using the NanoDrop system and a portion of RNA from each sample was diluted to equal concentrations. Real-time quantitative RT-PCR (qRT-PCR) analyses were performed using the iTaq Universal SYBR Green One-Step Kit (Bio-Rad, Cat# 1725151) with 5 ng RNA for each reaction. Primer sequences are summarized in Table S3. qRT-PCR was run using a CFX96 real-time PCR detection system thermal cycler (Bio-Rad). Data from qRT-PCR runs were read and analyzed using CFX Manager software (Bio-Rad).

### Transmission electron microscopy

Cells transduced with HA-tagged CAVIN1/PTRF or control viruses were washed twice with PBS and fixed for 30 min in buffer containing 2% glutaraldehyde, 4% paraformaldehyde and ruthenium red. They were scraped, centrifuged and the resulting pellet embedded in agarose for sectioning. Ultra-thin sections were cut with a microtome and collected on gold-coated grids. Imaging was performed using the HITACHI H-7600 TEM at the Oklahoma Medical Research Foundation Imaging Core Facility. Caveolae were identified on the plasma membrane by their characteristic 50 to 100 nm flask-shaped appearance. Caveolae numbers were quantified in 15 randomly selected and non-overlapping image fields at 15,000 to 20,000X from treatment groups. Quantification was performed on de-identified/masked images by an observer who did not know treatment group identity.

### Statistical analyses

Data are expressed as mean ± SD and statistical analyses were performed using GraphPad Prism eight software for the following tests: t-tests for paired and unpaired comparisons with only two groups; one-way ANOVA for multiple comparisons with a single variable; or two-way ANOVAs for experiments involving more than one variable. Post hoc tests included Tukey’s or Dunnett’s multiple comparison tests as indicated. Statistical significance was set at *p* < 0.05.

## Data availability

All data supporting the findings of this study are available within the article and its supporting information. Single-cell RNA sequencing data analyzed in this study are publicly available from previously published datasets (48, 52, 53, 54, 55, 56, 57). Raw data, additional microscopy images, and Western blot images are available from the corresponding author, Dr Michael Elliott (michael-elliott@ou.edu) upon reasonable request. The MIO-M1 cell line is available from the originating laboratory (G. Astrid Limb, University College London) upon a material transfer agreement.

## Supporting information

This article contains supporting information (48, 52, 53, 54, 55, 56, 57).

## Conflict of interest

The authors declare that they have no conflicts of interest with the contents of this article.
