# Supplementary material for: Non-caveolar caveolin - 1 in retinal Müller glia promotes innate immune responses
Source: J Biol Chem. 2026 Jun 23;302(8):113290. doi: 10.1016/j.jbc.2026.113290 (PMC13382776; doi:10.1016/j.jbc.2026.113290)
Supplement: Supporting Material [file mmc1.pdf]

Supplementary Information for

Non-caveolar caveolin-1 in retinal Müller glia promotes innate immune responses

Eric N. Enyong[1,2,Ŧ], Olawale O. Bankole[1,2,Ŧ], Jami M. Gurley[1], Mark E. McClellan[1], Libin Liu[3], Shi-Ying Ding[3], Martin-Paul Agbaga[2,4], Ana J. Chucair-Elliott[1,2], John D. Ash[5] and Michael H. Elliott[1,2,*]

[1]Department of Biochemistry & Physiology, [2]Department of Ophthalmology, Dean A. McGee Eye Institute, University of Oklahoma Health Campus, Oklahoma City, Oklahoma, [3]Department. of Biochemistry, Boston University School of Medicine, Boston, Massachusetts, [4]Department of Cell Biology, University of Oklahoma Health Campus, Oklahoma City, Oklahoma, [5]Department of Ophthalmology-UPMC Vision Institute, University of Pittsburgh School of Medicine, Pittsburgh, Pennsylvania

* **For correspondence:** Michael H. Elliott, michael-elliott@ou.edu

Ŧ Indicates equally contributing authors.

This PDF file includes:

Tables S1 to S3

Figures S1 to S10

Supplemental Table 1. Antibodies used in this study

Antigen	Vendor	Catalog No	Host Species	Application
CAVIN1/PTRF	Proteintech	18892-1-AP	Rabbit polyclonal	WB (1:1000)
CAVIN1/PTRF	Abcam	ab48824	Rabbit polyclonal	IHC (1:200)
CAV1	Cell Signaling	3267	Rabbit monoclonal (clone D46G3)	WB (1:1000)<br>IHC (1:200)
CAV1	BD Biosciences	610407	Mouse monoclonal (clone 2297)	IHC (1:100)
Tri-methyl-Histone H3 (Lys27)	Cell Signaling	9733	Rabbit monoclonal (clone C36B11)	WB (1:1000)
Histone H3	Cell Signaling	4499	Rabbit monoclonal (clone D1H2)	WB (1:1000)
HA-Tag	Cell Signaling	2367	Mouse monoclonal (clone 6E2)	WB (1:1000)<br>IHC (1:200)
GFP	Cell Signaling	2956	Rabbit monoclonal (clone D5.1)	WB (1:1000)
pIKKα (S176/180)	Cell Signaling	2697	Rabbit monoclonal (clone 16A6)	WB (1:1000)
Total IKKα	Cell Signaling	2682	Rabbit polyclonal	WB (1:1000)
pIKBα (Ser32)	Cell Signaling	2859	Rabbit monoclonal (clone 14D4)	WB (1:1000)
GS	Millipore	MAB302	Mouse monoclonal (clone GS-6)	WB (1:1000)<br>IHC (1:500)

GFAP	Millipore	MAB360	Mouse monoclonal (clone GA5)	WB (1:1000)
Vimentin	Abcam	8978	Mouse monoclonal (clone RV202)	WB (1:1000)
β-actin	Abcam	ab6276	Mouse monoclonal (clone: AC-15)	WB (1:1000)
Rabbit IgG	Sigma Aldrich	NA934-1ML	Donkey HRP conjugated	WB (1:5000)
Mouse IgG	Sigma Aldrich	NA931-1ML	Sheep HRP conjugated	WB (1:5000)
CD31	Dianova GmbH	DIA310	Rat monoclonal (clone SZ31)	IHC (1:50)
Mouse IgG	Invitrogen	A21202	Donkey, Alexa Fluor 488	IHC (1:500)
Mouse IgG	Invitrogen	A21125	Goat, Alexa Fluor 594	IHC (1:500)
Rabbit IgG	Invitrogen	A11008	Goat, Alexa Fluor 488	IHC (1:500)
Rabbit IgG	Invitrogen	A21207	Donkey, Alexa Fluor 594	IHC (1:500)
Rat IgG	Jackson ImmunoResearch	112-605-003	Goat, Alexa Fluor 647	IHC (1:500)
Rabbit IgG	Invitrogen	A32734	Goat, Alexa Fluor 680	Li-Cor WB (1:20,000)
Mouse IgG	Invitrogen	A32789	Donkey, Alexa Fluor 800	Li-Cor WB (1:5,000)

Supplemental Table 2. Taqman gene expression assays used in qPCR analyses

**gene/gene ID**	**Taqman Gene Expression assay ID**
CAV1	Hs00971716_m1 CAV1
CAV2	Hs00184597_m1 CAV2
PTRF	Hs00396859_m1PTRF
HPRT1	Hs2800695_m1 HPRT1

Supplemental Table 3. Primer sequence for qRT-PCR

**gene**	**forward primer (5'-3')**	**reverse primer (5'-3')**
IL-6	AAAGAGGCACTGGCAGAAAA	TTTCACCAGGCAAGTCTCCT
Actb	CTCTTCCAGCCTTCCTTCCT	AGCACTGTGTTGGCGTACAG
Tuba1a	GGCCAAGGTACAGAGAGCTG	CACGTTTGGCATACATCAGG

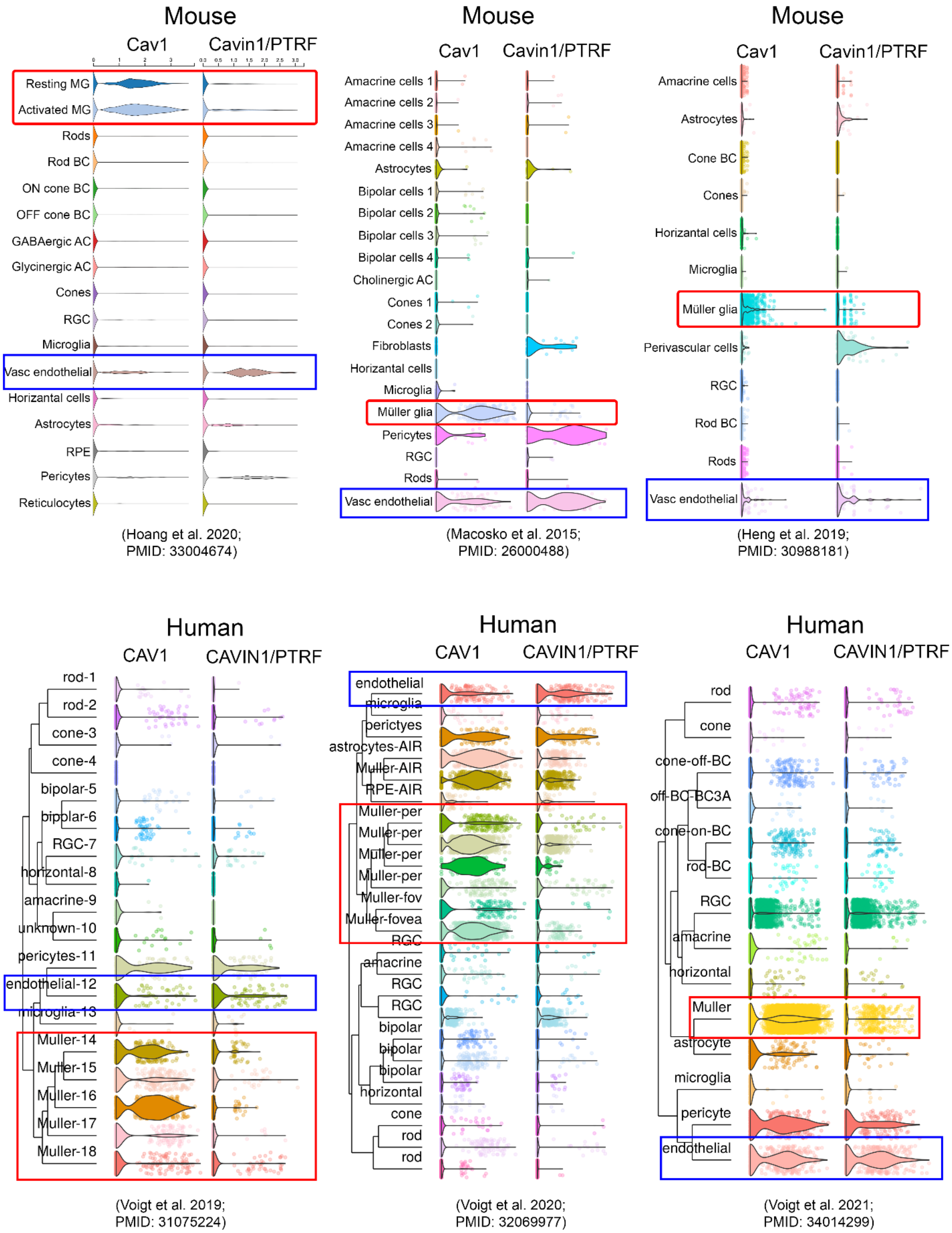

Fig. S1. Violin plots of cell type-specific expression of CAVIN1/PTRF and CAV1 in retinal single cell RNAseq data plotted from three separate mouse (1-3) and three separate human studies (4-6). In all studies, CAV1 is highly expressed in Müller glia (MG) populations (highlighted in red

boxes), as well as in vascular endothelium (highlighted in blue boxes) and pericytes (not highlighted). CAVIN1/PTRF is virtually undetectable in Müller glia in all studies, but expression is high in vascular endothelium and pericytes. Data plotted from Hoang et al.2020 can be accessed at https://proteinpaint.stjude.org/F/2019.retina.scRNA.html All other data were plotted using Spectacle https://singlecell.ivr.uiowa.edu/app/spectacle/ (7).

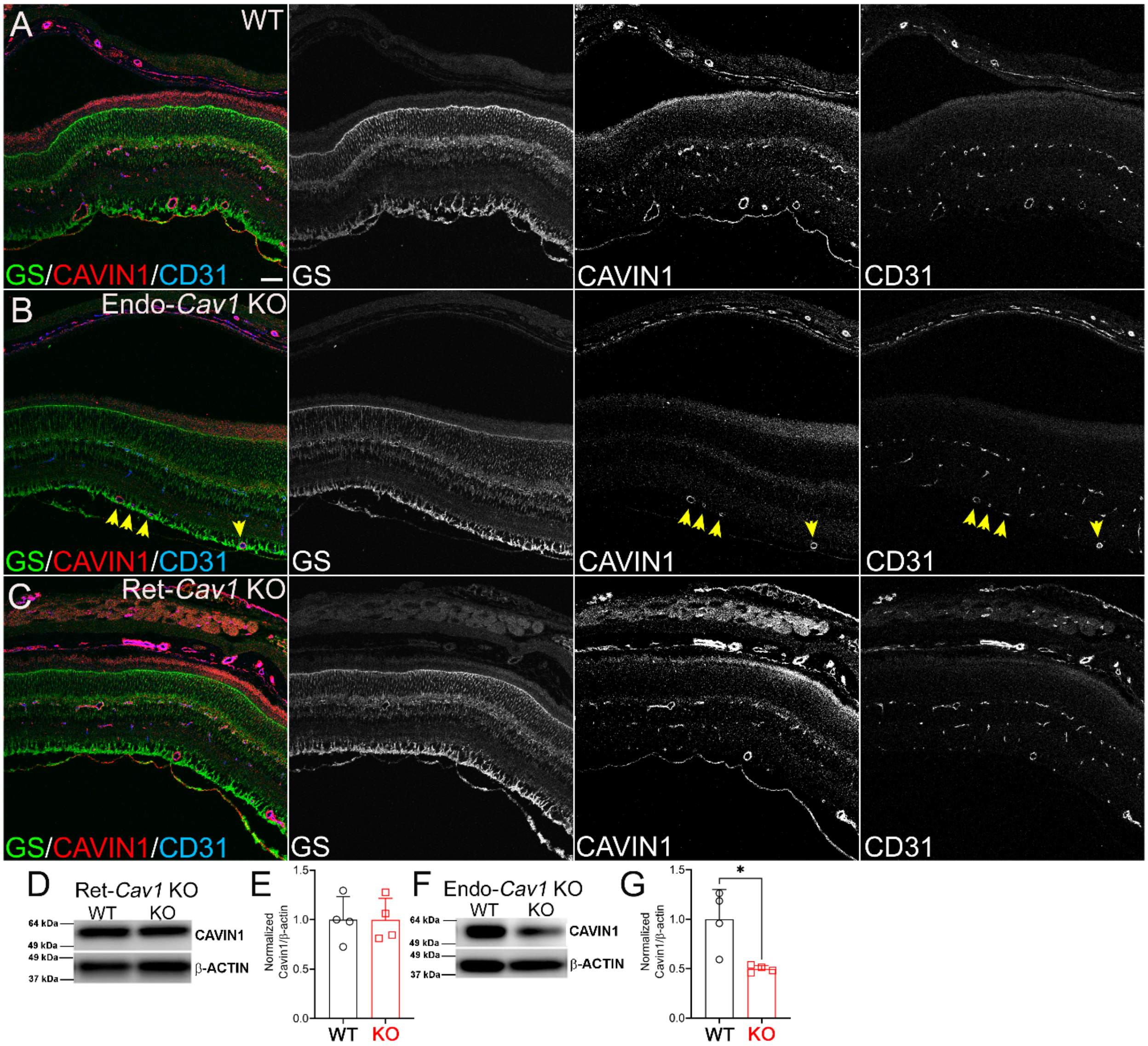

Fig. S2. CAVIN1/PTRF expression in the retina is predominantly from the vasculature and not the neuroretinal compartment. A) In WT mice, CAVIN1/PTRF co-localizes with CD31 to retinal and choroidal blood vessels and is virtually undetectable in the neuroretinal compartment. **B)** When *Cav1* is specifically ablated from the vascular endothelium (*Endo-Cav1-KO*), CAVIN1/PTRF immunoreactivity in retinal and choroidal vascular endothelium is dramatically reduced. Vessel associated immunoreactivity is mainly associated with vascular smooth muscle which is not targeted by the Tie2-Cre. **C)** When *Cav1* is specifically ablated from the neuroretinal compartment (including Müller glia) in retina-specific *Cav1* KO mice (*Ret-Cav1 KO*), CAVIN1/PTRF expression is unaffected. Scale bar = 50 µm. **D-G)** Representative Western blots and densitometric analyses confirming the IHC results. Retina-specific ablation of *Cav1* does not affect CAVIN1/PTRF protein levels in whole retinal extracts whereas endothelium-specific *Cav1* deletion significantly reduces CAVIN1/PTRF protein levels. These results provide additional support that CAVIN1/PTRF expression is localized to the retinal vasculature and is largely absent from Müller glia. (*$p < 0.05$; unpaired t-test)

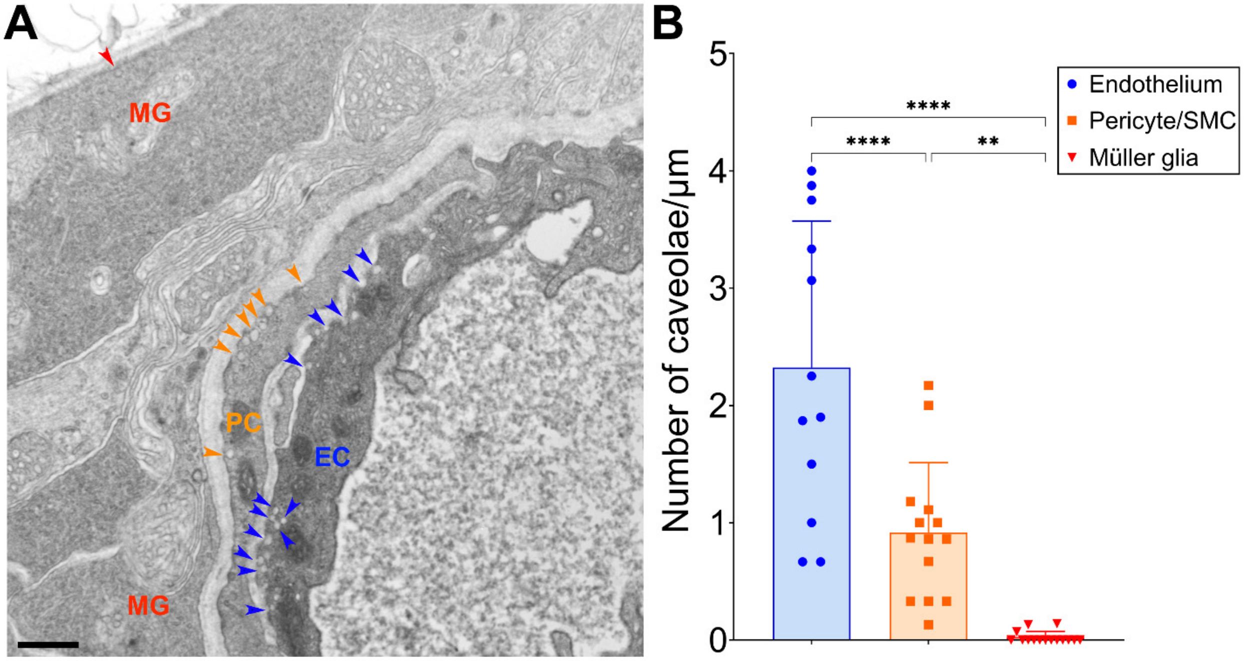

Fig. S3. Müller glia in mouse retina rarely form morphologically-identifiable caveolae. Representative TEM image showing distribution of caveolae in retinal vasculature but not in surrounding Müller glia (MG). Color-coded arrowheads label morphologically-identifiable caveolae in an endothelial cell (**EC**, **blue arrowheads**), a pericyte (**PC**, **orange arrowheads**), and a single caveola in a Müller glial cell (**MG**, **red arrowhead**). Caveolae were counted and normalized to membrane length in 17 TEM images were analyzed from n = 4 WT mouse retinas. Caveolae were abundant in EC and PC but were only rarely observed in MG. Scale bar = 500 nm.

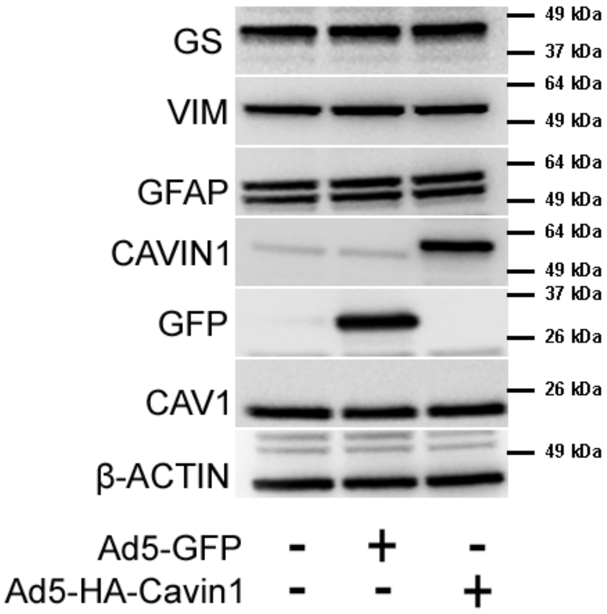

Fig. S4. Authentication of MIO-M1 Müller glia. MIO-M1 cells under our culture conditions express Müller glial markers glutamine synthetase (GS), vimentin (Vim), and glial fibrillary acid protein (GFAP) and abundantly express CAV1 with very little CAVIN1 expression. CAVIN1 or GFP are abundantly expressed in MIO-M1 Müller glia after transduction with Ad5-HA-CAVIN1/PTRF or Ad5-GFP, respectively.

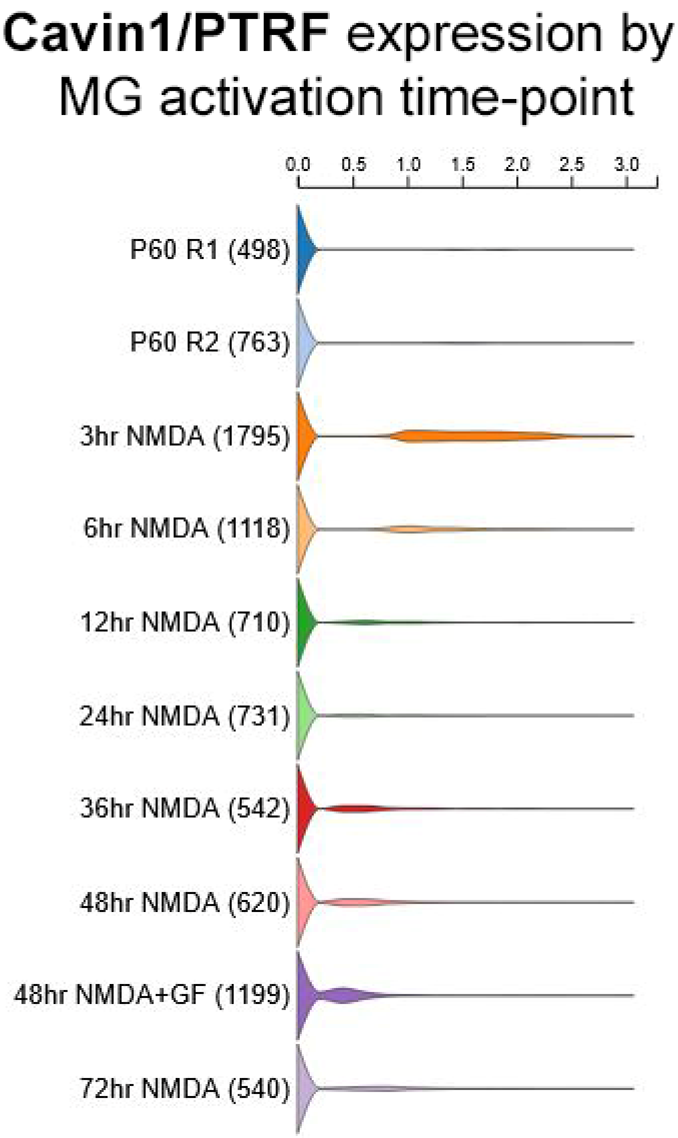

Fig. S5. Violin plots showing time-dependent upregulation of CAVIN1/PTRF transcript in Müller glia (MG) in retinal single cell RNAseq data plotted from (1). As shown, CAVIN1/PTRF RNA expression peaks at 3 hours after NMDA excitotoxic insult to the retina.

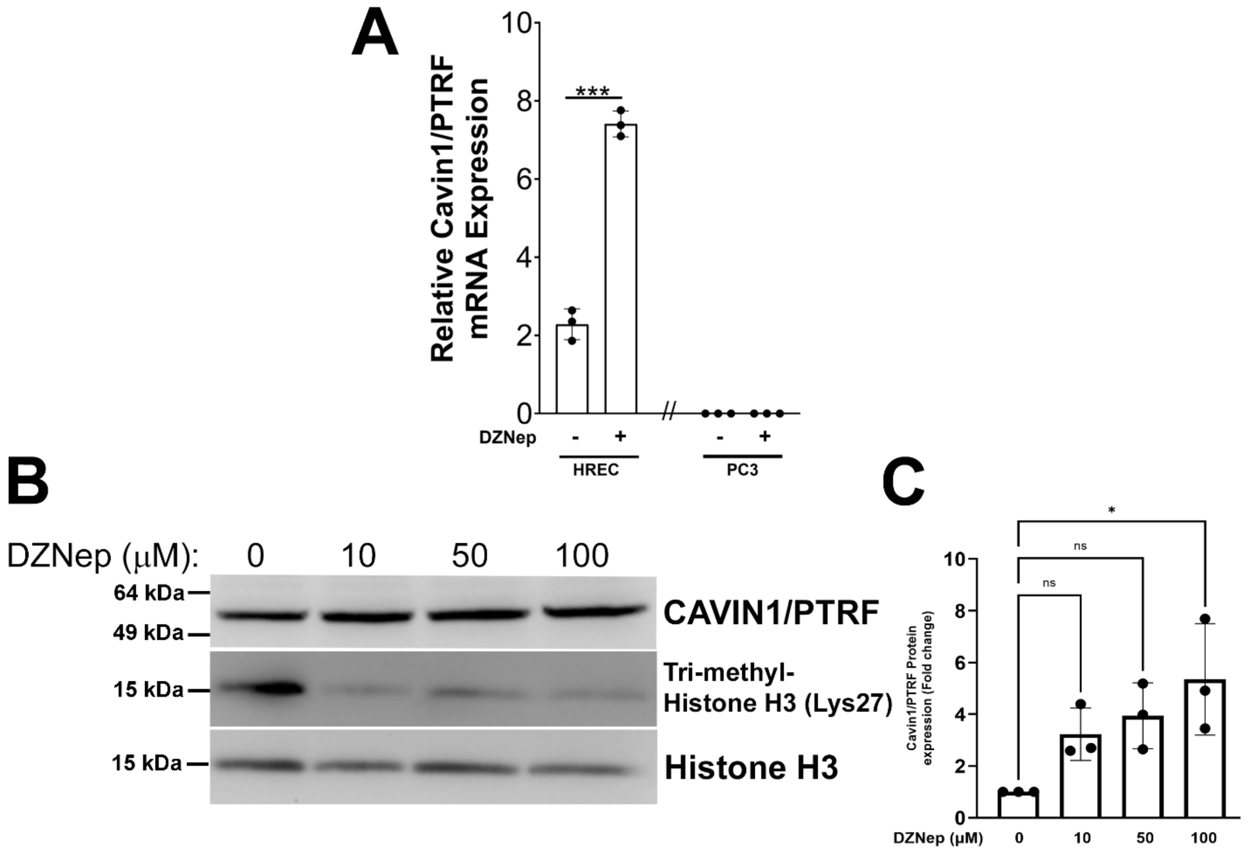

Fig. S6. Inhibition of EZH2 histone methyltransferase induces CAVIN1/PTRF expression in MIO-M1 Müller glia, Human Retinal Endothelial Cells (HRECs) but not in PC3 (Prostate Cancer) cells. **A)** qRT-PCR analysis showing significant induction of CAVIN1/PTRF mRNA expression in HREC 48 h after treatment with 100 μM DZNep. CAVIN1/PTRF mRNA was undetectable in PC3 cells aven after 48 h of 100 μM DZNep treatment in agreement with data indicating a null mutation in this cell line. B) Representative Western blots and C) densitometric analysis showing DZNep-induced reduction of tri-methylated Histone (H3) and dose-dependent induction of CAVIN1/PTRF protein in HREC that reached significance at 100 μM DZNep. Cells were treated with 0, 10, 50 or 100 μM of DZNep for 48 h. Total Histone (H3) was used as loading control (n=3, One-way ANOVA with Dunnett’s multiple comparison tests, *$p < 0.05$).

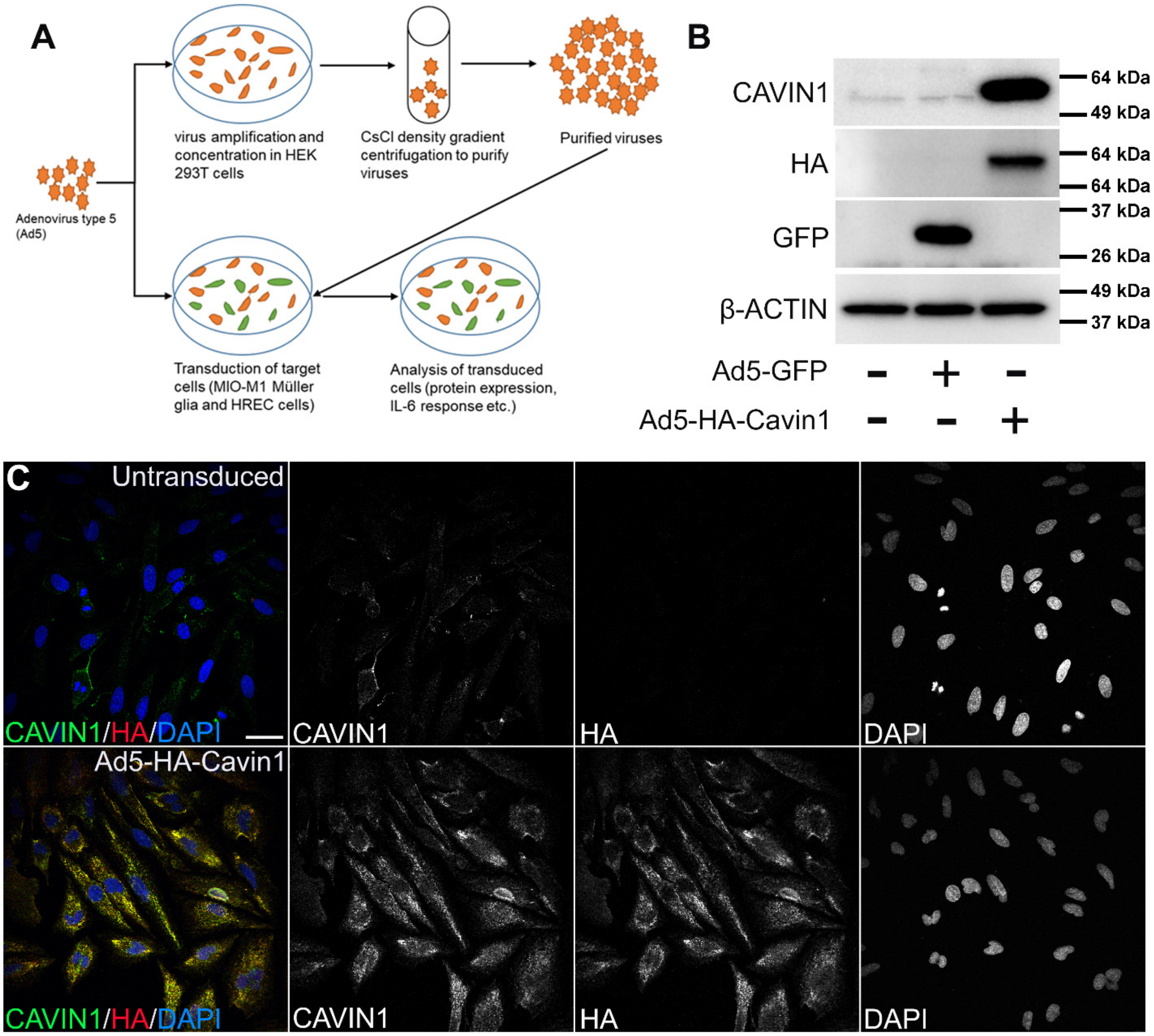

Fig. S7. Ad5-mediated expression of HA-tagged CAVIN1/PTRF in MIO-M1 Müller glia. A) Schematic diagram illustrating the workflow for expressing HA-tagged CAVIN1/PTRF in MIO-M1 Müller glia, mediated by adenovirus serotype 5 (Ad5). **B)** Western blots showing that MIO-M1 Müller glia transduced with Ad5-HA-CAVIN1/PTRF expressed a protein detected by both HA-tag and CAVIN1 antibodies. **C)** Immunostaining of MIO-M1 Müller glia showing expression and co-localization of HA and CAVIN1/PTRF proteins after transduction with HA-tagged CAVIN1/PTRF virus (Ad5-HA-CAVIN1). Endogenous CAVIN11/PTRF is only weakly expressed in MIO-M1 Müller glia. Scale bar = 50 μm.

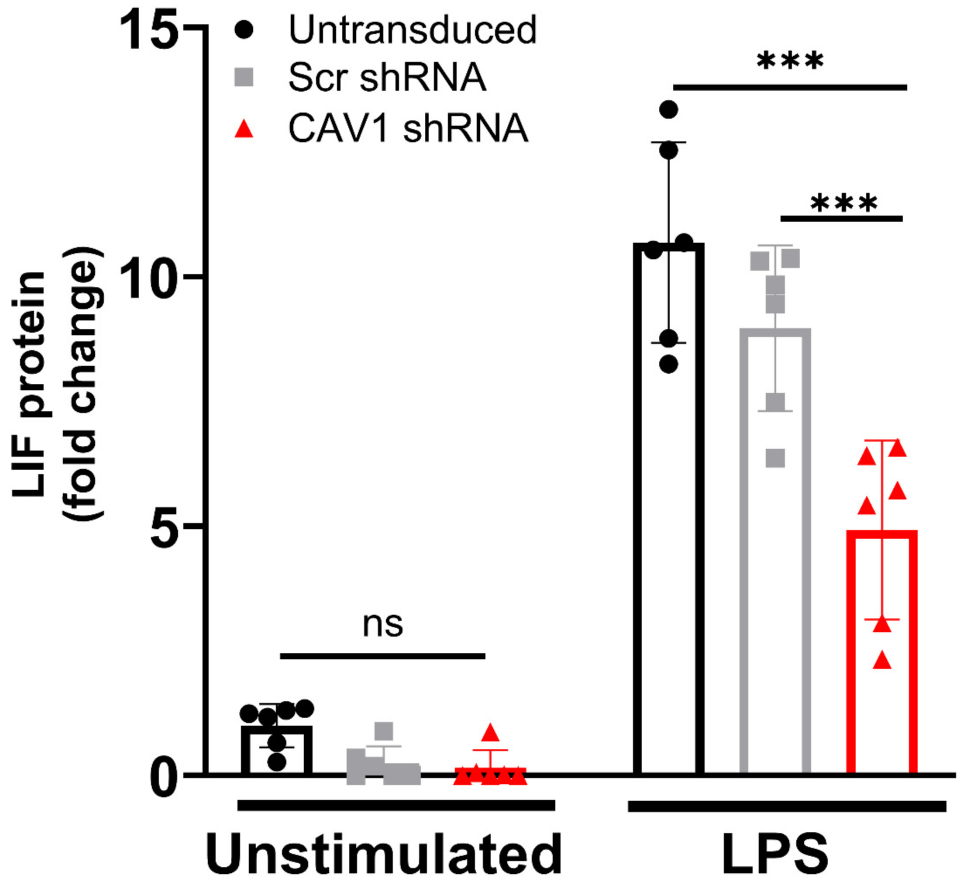

Fig. S8. Silencing non-caveolar CAV1 in MIO-M1 Müller glia reduces TLR4-mediated LIF induction. Cells were either untransduced or transduced with Ad5-Scr control shRNA or Ad5-CAV1-shRNA viruses for 96 h, followed by stimulation with 0.02 μg/ml LPS. After 24 h, tissue culture media was harvested to measure LIF levels by ELISA (Two-way ANOVA with Tukey's post hoc test for CAVIN1/PTRF transduction effect *P < 0.05).

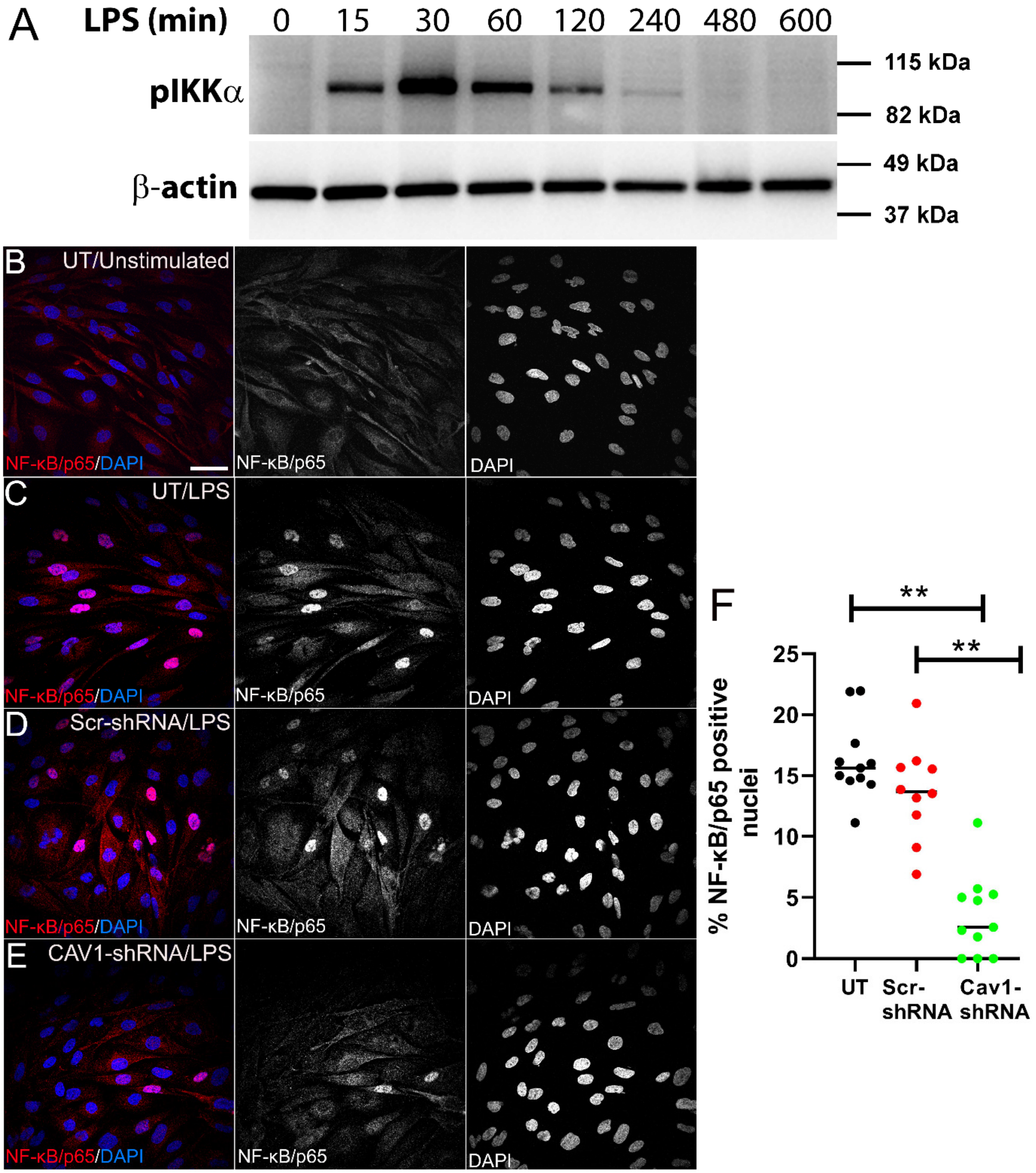

Fig. S9. LPS induces NF-κB signaling in Müller glia and activation is suppressed by silencing CAV1 expression. A) LPS stimulation results in time-dependent phosphorylation of IKKα in MIO-M1 Müller glia, peaking at 30 min. β-actin was used as loading control. **B-E)** CAV1 silencing reduces LPS-stimulated NF-κB/p65 nuclear localization in MIO-M1 cells. MIO-M1 cells were either untransduced or transduced with Ad5-control or Ad5-CAV1-shRNA viruses for 96 h, and then stimulated with 0.5 μg/ml LPS for 4 h. Cells were rinsed and processed for immunocytochemistry. Scale bar = 50 μm. **F)** Percentage of NF-κB/p65-positive nuclei is significantly reduced in CAV1-silenced cells. Each data point represents the quantification from non-overlapping image fields (One-way ANOVA with Tukey's multiple comparison, *$p < 0.05$).

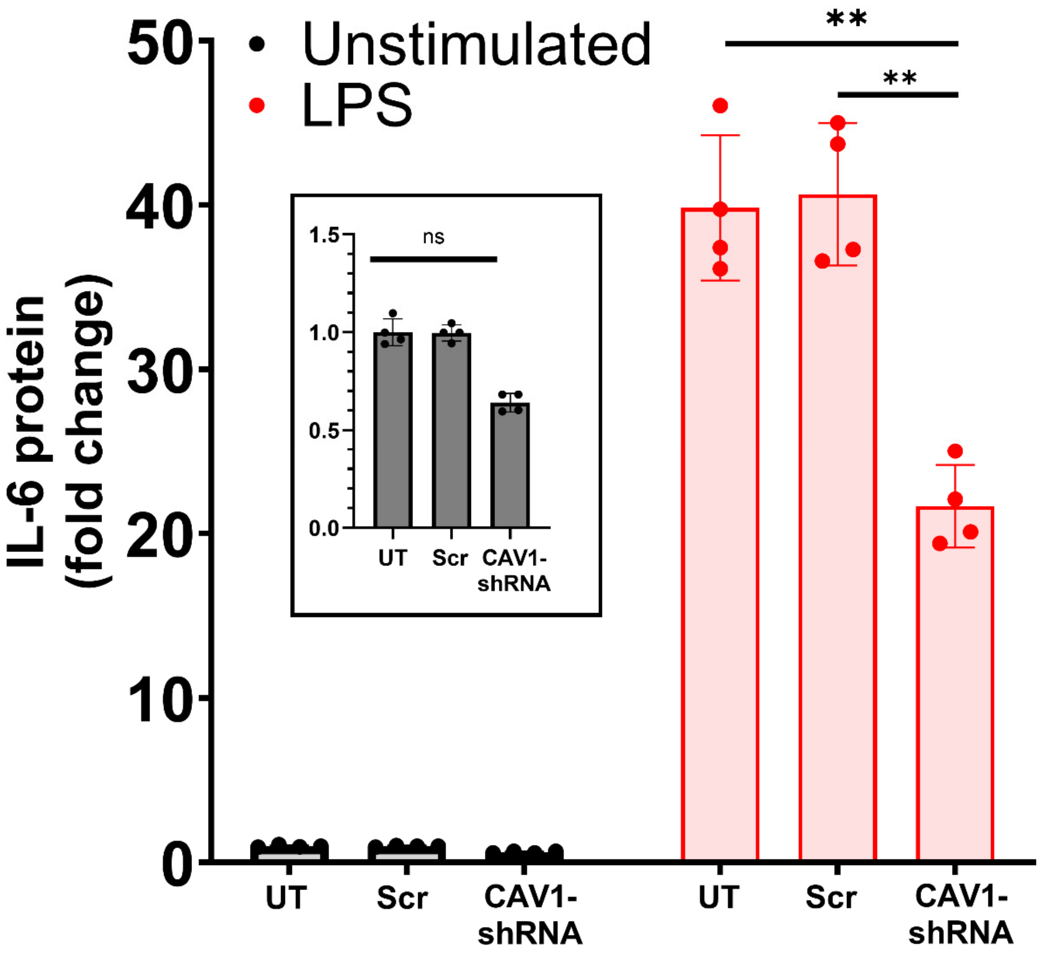

Fig. S10. CAV1 silencing reduces intracellular IL-6 in MIO-M1 Müller glia cell lysates. MIO-M1 Müller glia were transduced with CAV1-shRNA, control virus with scrambled shRNA, or left untransduced. After 96 h, cells were stimulated with 0.02 µg/ml LPS for 6 h. Cell lysates were harvested after washing extensively with PBS and intracellular IL-6 levels were measured by ELISA. CAV1 silencing significantly suppressed TLR4-mediated IL-6 response when compared to control shRNA and untranduced control (Two-way ANOVA for LPS stimulation effect, $*p<0.05$; CAVIN1/PTRF transduction effect, $*p< 0.05$). There was a small but not significant decrease in basal IL-6 levels in CAV1-silenced MIO-M1 cells without LPS stimulation (inset). UT: untransduced, Scr: scrambled-shRNA, Cav1: CAV1-shRNA

Supplement References

1. Hoang, T., Wang, J., Boyd, P., Wang, F., Santiago, C., Jiang, L., Yoo, S., Lahne, M., Todd, L. J., Jia, M., Saez, C., Keuthan, C., Palazzo, I., Squires, N., Campbell, W. A., Rajaii, F., Parayil, T., Trinh, V., Kim, D. W., Wang, G., Campbell, L. J., Ash, J., Fischer, A. J., Hyde, D. R., Qian, J., and Blackshaw, S. (2020) Gene regulatory networks controlling vertebrate retinal regeneration. *Science* **370**, eabb8598
2. Macosko, E. Z., Basu, A., Satija, R., Nemesh, J., Shekhar, K., Goldman, M., Tirosh, I., Bialas, A. R., Kamitaki, N., Martersteck, E. M., Trombetta, J. J., Weitz, D. A., Sanes, J. R., Shalek, A. K., Regev, A., and McCarroll, S. A. (2015) Highly Parallel Genome-wide Expression Profiling of Individual Cells Using Nanoliter Droplets. *Cell* **161**, 1202-1214
3. Heng, J. S., Rattner, A., Stein-O'Brien, G. L., Winer, B. L., Jones, B. W., Vernon, H. J., Goff, L. A., and Nathans, J. (2019) Hypoxia tolerance in the Norrin-deficient retina and the chronically hypoxic brain studied at single-cell resolution. *Proceedings of the National Academy of Sciences of the United States of America* **116**, 9103-9114
4. Voigt, A. P., Whitmore, S. S., Flamme-Wiese, M. J., Riker, M. J., Wiley, L. A., Tucker, B. A., Stone, E. M., Mullins, R. F., and Scheetz, T. E. (2019) Molecular characterization of foveal versus peripheral human retina by single-cell RNA sequencing. *Experimental eye research* **184**, 234-242
5. Voigt, A. P., Binkley, E., Flamme-Wiese, M. J., Zeng, S., DeLuca, A. P., Scheetz, T. E., Tucker, B. A., Mullins, R. F., and Stone, E. M. (2020) Single-Cell RNA Sequencing in Human Retinal Degeneration Reveals Distinct Glial Cell Populations. *Cells* **9**
6. Voigt, A. P., Mullin, N. K., Whitmore, S. S., DeLuca, A. P., Burnight, E. R., Liu, X., Tucker, B. A., Scheetz, T. E., Stone, E. M., and Mullins, R. F. (2021) Human photoreceptor cells from different macular subregions have distinct transcriptional profiles. *Human molecular genetics* **30**, 1543-1558
7. Voigt, A. P., Whitmore, S. S., Lessing, N. D., DeLuca, A. P., Tucker, B. A., Stone, E. M., Mullins, R. F., and Scheetz, T. E. (2020) Spectacle: An interactive resource for ocular single-cell RNA sequencing data analysis. *Experimental eye research* **200**, 108204
